# Compensation incentives and heat exposure affect farm worker effort

**DOI:** 10.1371/journal.pone.0259459

**Published:** 2021-11-02

**Authors:** Qianyao Pan, Daniel A. Sumner, Diane C. Mitchell, Marc Schenker

**Affiliations:** 1 Agricultural and Resource Economics, University of California, Davis, California, United States of America; 2 Member of Giannini Foundation of Agricultural Economics, Davis, California, United States of America; 3 Department of Public Health Sciences, University of California, Davis, California, United States of America; Vietnam National University, VIET NAM

## Abstract

Farm workers are exposed to high risk of heat-related illness, especially when their jobs require working outside at a fast pace during hot days. Climate change has increased the number of days with high temperatures, and thereby the amount of time that farm workers are likely exposed to extreme heat. To better understand how high heat exposure affects farm workers, this study investigates how crop workers respond to heat exposure and estimates the effects of different pay and work arrangements on workers’ responses to heat exposure. We explore, specifically, whether piece-rate arrangements increase workers’ effort during periods with high heat exposure compared to workers paid by hourly wages. We use observational data from detailed measurements of localized heat exposure and individual workers’ effort in the field. First, these results show workers adjust their effort in response to heat exposure when the heat exposure level changes. Second, piece-rate arrangements increase workers’ effort during work shifts. Third, piece-rate arrangements allow workers to modify their effort more easily during different heat exposure levels. When facing low levels of heat exposure, workers who were paid by piece-rate arrangements exert a higher effort than workers paid by hourly wages, up until WBGT is 26.6˚C. When facing high levels of heat exposure (with WBGT exceeding 29.6˚C), workers paid by piece-rate arrangements lower their effort compared to workers paid by hourly wage arrangements.

## 1. Introduction

Climate change will cause an increasingly unsafe working environment for farm workers given the current work arrangements. U.S. farm workers have high risk of illnesses related to heat stress, with twenty times more risk of fatality from heat strain than average U.S. civilian workers [1]. Tigchelaar, Battisti and Spector [2] find that climate change will likely worsen the farm worker’s working conditions by increasing the number of days with extreme heat. They predict that by the end of 2050 the number of days with extreme heat will double for U.S. farm workers.

The hired farm workforce in the United States consists primarily of low-income Latino immigrants. Most farm workers have low levels of education, live in poor housing conditions, and have less access to healthcare and legal protections than do most U.S. workers [3]. Based on the 2015–2016 National Agricultural Worker Survey (NAWS), nearly half of all farm workers had no work authorization to work in the U.S. The average level of education completed by farm workers was eighth grade. About 68 percent of the farm workers were male. More than half of the farm workers interviewed in NAWS had no medical insurance of any kind.

Farm workers who work in fields, the subject of this study, are among the most vulnerable laborers to heat exposure [4]. Some farm production practices, such as thinning and harvesting, require intensive labor activity from farm workers when there is likely to be significant heat exposure. For example, many fruits and vegetables require significant hand labor during harvesting, which is often during the hottest season of the year. These concerns are growing with climate change that increases the potential heat exposure for those working outside during hot summer months. Therefore, it is increasingly important to understand how different working environments, such as payment arrangements, affect heat exposure and potential illness.

## 2. Background

Recent literature finds occupational heat exposure cause an immediate heat strain symptoms and heat-related illness which ranges from heat rash to more severe heat stroke [4–7]. These studies find that the impact of heat exposure on workers’ health differs across work intensity. For example, workers with an intense physical activity at work are more likely to show heat strain symptoms under the heat exposure than workers with light physical demands at work.

Work schedule, shift rules, pay arrangements, and worker and manager responses, may affect how and how much workers are exposed to extreme heat and the potential for heat-related illness. Sometimes, there may be few alternatives to working during periods of extreme heat, such as harvests that must be completed in the fields during a specific timeframe in the hot summer months. However, by arranging to work early in the day and avoiding working outside during the peak heat period, farm managers and workers can help reduce heat exposure and lower their risk of heat-related illness. Moreover, work rules that encourage workers to take more breaks and work at a less strenuous pace when it is very hot can help reduce workers’ risk of heat-related illness when there is high heat exposure. While there are regulations that help manage workers heat stress and heat related illness, studies find that risk factors, such as gender, environmental heat exposure, and Body Mass Index (BMI), contribute to the risk of heat related illness [8].

This paper focuses on work arrangements and the incentives facing and the responses of hired farm workers. Farm workers may expose themselves to higher risk of heat-related illness when the incentive of increased income encourages working at a fast pace even when there is high heat exposure. The impact may depend on how pay is determined. Financial arrangements by farm operators and farm employees may both encourage and provide incentives for longer hours per day and fewer breaks, even on hot days. Farm operators and managers may expose themselves and workers to higher risks of heat-related illness, even when they may know of the risks of heat-related illness. Likewise, hired farm workers may face strong financial incentives to work strenuously even during hot times of the day and take fewer breaks than might be recommended.

### 2.1 Pay arrangements, incentives, heat and productivity

Three pay arrangements are common for hired farm workers. First, some workers, often managers, supervisors or specialists, are paid a fixed monthly or weekly salary. The amount of earnings is independent of how much of any specific tasks are accomplished, although job performance evaluations of the worker do reflect some metrics of the amount and quality of work. This payment arrangement is uncommon among field workers.

Second, field workers are often paid an hourly wage rate, which is one of the pay arrangements studied in this paper. Such workers are required to work during specific hours of the day and are paid a wage rate per hour. Specific meal breaks and short rest breaks are specified and may be required under labor law and regulations.

A third common pay arrangement for hired farm field workers is payment based on the amount of a specific task that is accomplished. Such piece-rate pay arrangements occur when the output of the tasks can be readily measured, and workers can therefore be readily and conveniently paid by the amount of measured output. Piece-rate arrangements are common for tasks such as pruning or thinning, when workers are paid by the row, tree or vine. Piece rates are also common for harvesting when workers are paid by the volume or weight of the product harvested.

Piece-rate arrangements allow the farm operator to pay farm workers directly for the task accomplished, allow workers to earn different amounts based on their skills and efforts, and create a financial incentive for workers to work intensively to achieve higher income during a day, a week or a season. Piece-rate arrangements may also create short-run incentives to work intensively, even when heat stress may become a factor. Pay arrangements may also vary for individual farmworkers over the growing season.

Several studies report that piece-rate payments are associated with different risks of heat-related illness for crop workers [9–12] and occupational injury [13]. Spector et al. [9] finds farm workers paid by piece-rate arrangements in harvesting and thinning tasks in Central and Eastern Washington State are more likely to have self-reported heat-related illness symptoms than workers paid by hourly wages. Moyce et al. [10–12] shows that heat strain and piece-rate work were associated with acute kidney injury for agricultural workers, which will lead to long term kidney damage. These articles also note that piece-rate arrangements incentivize workers to increase effort and take fewer breaks for rest, hydration, and restroom use. Early-stage symptoms for heat-related illnesses (such as dehydration and dizziness) usually do not require treatment when workers adjust their work intensity in response by resting and drinking water [14]. Spector et al. [9] identify the risk factors for heat-related illness using workers’ self-reported symptoms, i.e., dizziness, light-headedness or heavy sweating. Workers can mitigate the symptoms by taking rests or water intake. Johansson et al. [13] reviewed 31 studies summarizing the association between piece-rate payments and occupational injury and 27 studies found workers paid by piece-rate arrangements are associated with a higher rate of accidental injuries. However, these studies do not document causality impacts of piece-rate arrangements on occupational injury. For example, workers drawn to piece rate jobs, say because they are more than likely to rush on the job, may be the same workers who are prone to injury on the job because they may be less careful.

In contrast to the potential risk factors that piece-rate pay arrangements may bring to workers, Wadsworth, Courville and Schenker [15] found that farm workers showed preferences for a piece-rate pay arrangement because it allows the workers to take more control of their working conditions.

Much economic research has linked piece-rate arrangements to increases in productivity in a variety of models that examine the role of measurement and information cost. Well-known empirical studies have documented piece rate impacts on productivity [16,17]. Studies indicate that agricultural workers paid by piece-rate arrangements have higher productivity than workers paid by other pay arrangements [16,17].

There are relatively few research papers on the relationship between heat exposure and agricultural worker effort and productivity. For non-agricultural workers, studies find nonlinear relationships between temperature and productivity for office workers [18], and Indian manufacturing workers [19]. For agricultural workers, Sahu, Sett and Kjellstrom [20] find that heat exposure decreases work productivity for Indian rice harvesters. However, their work was criticized for omitting confounding worker demographic variables which result in differences in productivity by Quiller et al. [21]. In the study, Quiller et al. [21] find no statistically significant response of orchard workers’ productivity to heat exposure after controlling worker demographic variables. As noted, with climate change, the issue of heat exposure for farm workers is likely to become more significant and requires additional research interest.

### 2.2 Climate change and farm workers

Climate change will likely increase the heat exposure that farm workers face. According to Tigchelaar, Battisti and Spector [2], climate projections envision an increase in the number of days with extreme heat. According to the Fourth National Climate Assessment, the mean temperatures are projected to be 1.6°C higher for the period 2021–2050 relative to 1976–2005. Daily extreme temperatures are projected to increase substantially causing an increase of 20 to 30 days per year with a maximum temperature over 32°C in most areas in the U.S. by 2050. Therefore, health concerns related to high levels of heat exposure are growing with climate change. It is now even more important to understand how heat exposure affects farm workers’ health and their work intensity.

## 3. Methods

This section explains the survey data and metrics used to describe the heat exposure and employment situation of our sample of hired farm workers. It also explains the statistical methodology to test hypotheses and specifically to identify causation in the regression specifications.

### 3.1 Survey data and metrics

We analyzed data on hourly temperature and workers’ effort for 575 field workers from 31 farms in Central and Imperial Valleys in California for 82 days during the summer of 2014 and 2015. The survey collected information on workers for one day each, with no worker in the sample for more than one day. We have data on more than 6650 worker-hours with the information of their effort level and heat exposure by the hour. For each worker and job on each day, we also have the payment arrangements, task type, hire type (contractor employee or direct hire), and several demographic characteristics including gender, Body Mass Index (BMI), and age. We have no data on how much the farm workers were paid. Piece-rate payments typically differ across tasks and commodities. The piece rates are usually calibrated such that productive workers are paid more than the hourly wage rate available for all workers. To get a sense of the scale of wage rates, we note that San Joaquin Valley farm workers who were engaged in raisin winegrape harvesting and pruning tasks were paid 12 USD per hour in the Central Valley in California in 2016 [22].

The dataset was collected in a series of field studies, including observational data, data from interviewer-administered questionnaires, wearable data logging devices and weather station records. The field studies were conducted as part of the California Heat Illness Prevention Study (CHIPS). The detailed data collecting procedure can be found in Mitchell et al. [23]. The University of California, Davis Institutional Review Board approved the CHIPS protocols. Workers provided written consent before participating in CHIPS.

The heat exposure in our study is measured as the Wet Bulb Globe Temperature (WBGT). The Wet Bulb Globe Temperature (WBGT) is the main measurement of environmental heat exposure following the suggestion by National Institute for Occupational Safety and Health (NIOSH). During the field study, the WBGT was measured using a QUESTemp 36 environment monitor on a 1.2-meter tripod (Quest Technologies, Inc., Oconomowoc, WI) placed in the same field as the workers. The worker effort is measured in metabolic equivalents (METs) that is energy expenditure in units of 1000 calories per hour. The metabolic equivalents were measured using an accelerometer (Actical^TM^ Philips Respironics, Murrysville PA). Workers wore these accelerometers on their waist belt during their shift. The accelerometers recorded workers’ minute level activity counts, with breaks being recorded with value zero. The hourly metabolic rate is the average of the minute level metabolic rate computed from the minute level activity counts. The hourly metabolic rates differ across crops and tasks. Workers with tasks that mostly involves arm movements, such as sorting, and pruning vines, have lower metabolic rates than workers engaged in tasks that involved climbing ladders, for example. See Table S1 and S2 in S1 Appendix for the average hourly metabolic rate distribution across tasks and crops. Readers can find more detailed information regarding the computation of WBGT and METs and data collecting in the supplemental material.

### 3.1 Linear regression model to identify the impact of piece-rate arrangements

Consider a linear model to specify workers’ effort as a function of wage policy and heat exposure in workplace:

$$mets_{ih}=\beta_{0}+\beta_{1}PieceRate_{i}+\beta_{2}wbgt_{ih}+\beta_{3}wbgt^{2}+(\beta_{4}wbgt_{ih}+\beta_{5}wbgt_{ih}^{2})\cdot PieceRate_{i}+\beta_{6}Hour+\beta_{7}Hour^{2}+\beta X_{i}+\epsilon_{ih}$$ (1)

where the subscript i indicate an individual worker, the subscript h indicates hour of the day. The outcome variable *mets*_*ih*_ is the metabolic equivalents which measures worker i’s effort as metabolic equivalents at hour h. The variable *PieceRate*_*i*_ is one if worker i receives a piece-rate wage and zero if worker i receives an hourly wage for the observed shift. The variable *wbgt*_*ih*_ is Wet Bulb Globe Temperature (WBGT) that measures heat exposure of worker i at hour h (see S1 Appendix for the definition of WBGT). A set of variables *X*_*i*_ is a vector of worker i’s characteristics (age, gender, and BMI) and job characteristics (hire type, tasks, and months), and a quadratic hour of day variable. The variable *ε*_*ih*_ is an error term.

The parameters β_1_ estimate the average differences of effort between workers paid by piece-rate arrangements and hourly wages, conditional on heat exposure. The parameters *β*_*2*_ and *β*_*3*_ estimate the average differences of effort when the heat exposure increases 1˚C from the mean for workers paid by hourly wage. The *β*_*4*_ and *β*_*5*_ estimate the differences of workers’ response between workers paid by piece-rate arrangements and hourly wage in effort when the mean heat exposure increases 1˚C from the mean. The parameter β_4_ specifies the piece-rate wage’s first-order effects while the parameter β_5_ specifies the piece-rate wage’s second-order effects on worker’s response to heat exposure. By this decomposition, we can check whether the effects of the piece-rate wage policy on workers’ effort increase or decrease as heat exposure becomes more severe.

The impact of piece-rate arrangements on worker’s effort is *β*_*1*_*+β*_*4*_*wbgt*_*ih*_*+β*_*5*_*wbgt*_*ih*_^*2*^, which is a quadratic function of heat exposure. The marginal response of hourly-waged workers to heat exposure is *β*_*2*_*+2β*_*3*_*wbgt*_*ih*_ while the marginal response of workers paid by piece-rate arrangements to heat exposure is *β*_*2*_*+β*_*4*_*+2(β*_*3*_*+β*_*5*_*) wbgt*_*ih*_.

We use the hours of the day to capture changes in effort over the time of the shift in the workplace. For example, workers may be less active during early morning when they are not familiar with the specific work situation or are still warming up to the tasks and later of the day due to fatigue. A quadratic hours of day variable captures nonlinear responses of workers’ effort to the hours of the day.

### 3.2 Propensity score matching to reduce selection bias

The sample of workers who are paid by piece-rate arrangements may be self-selected such that workers who chose jobs that are paid by piece-rate arrangements (or are chosen by employers) are different from workers who chose to work on jobs that are paid by hourly wages. These underlying differences, such as differences in unobserved worker characteristics between the two groups, may also lead to different responses to high heat exposure. Directly comparing the responses of the two groups of workers, in terms of their responses to heat and their effort, may give a biased estimate of the causal impacts of piece-rate arrangement (selection bias).

In this study, we apply propensity score matching to control the potential selection bias in estimating causal parameters. The propensity score matching method uses a sub-sample constructed to avoid selection bias and estimates parameters of interest on that sub-sample. The probability of selection into a treatment, in this study which is the probability of being paid by piece-rate arrangements, is the propensity score. The propensity score plays an important role in selection bias control and in matching [24,25].

The first step of our propensity matching procedure is to estimate the probability of a worker being paid by a piece-rate pay arrangement given the workers’ observational characteristics. In this analysis, we use a logistic regression using the 244 workers who conduct tree pruning/thinning and harvesting. We use the 244 workers to estimate the propensity score matching because workers with other tasks (irrigation, equipment driving, and rake and hoe) were rarely paid by piece-rate arrangements given the difficulty of measuring or monitoring the work output. Hence, in the logistic regression that predicts piece rate participation, we include only workers whose tasks are pruning/thinning or harvesting where we have enough observations to match workers more precisely especially in terms of tasks. The logistic regression is shown below as the Eq (2) in the following:

$$PieceRate_{i}=logit(\delta_{0}+\delta_{1}Female_{i}+\delta_{2}BMI_{i}+\delta_{3}Age+\delta_{4}Contractor_{i}+\sum_{k=6}^{10}\delta_{5k}{Month}_{ik}+\sum_{j=1}^{3}\delta_{6j}Task_{ij}+\xi_{i})$$ (2)

where the subscript *i* indicates an individual worker, the subscript *k* indicates the month of the year, and the subscript *j* indicates the type of tasks. The outcome variable *PieceRate*_*i*_ is the binary variable of the worker *i*, with value 1 being paid by piece rate. The variable *Female*_*i*_ is one if the worker is female, *BMI*_*i*_ measures the workers Body Mass Index (BMI), *Age*_*i*_ is the worker’s age, *Contractor*_*i*_ equals one if the worker is employed by a farm labor contractor (and zero if worker is employed directly by the farm). The month variables and task variables indicate fixed effects of each of the five months and each of the two types of tasks. The variable *ξ*_*i*_ is an error term. We used 243 workers (out of 244 workers) who are operating tree pruning or thinning and harvesting to estimate the coefficients, with one worker having no employer type formation.

Once the predictions from the logistic regression are completed, the second step of propensity matching is to “match” each worker who is paid by a piece-rate arrangement to a “similar” worker paid by hourly wages. The matching algorithm uses the projected probability of being paid by piece rate (propensity score) as a distance value, and matches the workers actually paid by piece rate with an hourly paid worker based on the propensity score. The algorithm matches piece rate workers following the order of propensity score from largest to smallest values. Some workers paid by hourly wage arrangements are discarded before the matching step due to their tasks, such as irrigation, or supervision for which no piece rate is ever observed. All workers but one (with missing information on hiring type) who are paid by piece-rate arrangements are included in the matching step. After matching, we select all the matched workers and then run regression specification in Eq (1) on the matched sample. We carried out robustness checks on different data matching approaches. For example, instead of the propensity matching step, we select tasks for which both hourly wages and piece-rate pay arrangements were present and then run the regression specification in Eq (1). We also conduct robustness checks on using air temperature as heat exposure measure, and allow separate regression estimates for male and female workers. The methods and results are discussed in the S1 appendix.

## 4. Results

This section first describes data used in detailed econometrics with emphasis on the patterns of temperatures and tasks across workers and across time periods during the workdays. We then present the estimated effects of piece-rate pay arrangements on worker effort, with emphasis on implications for potential heat stress. To establish robustness of the estimates, we provide regression parameter estimates without using a propensity score technique. Further documentation of robustness of the results are in the appendix.

### 4.1 Data summary

In our data, the average age of workers is about 39 years old, and the average BMI is slightly greater than 25 (the cutoff for overweight). There are a total 378 (65.7%) male workers and 197 (34.3%) female workers among the 575 workers. About 268 workers are directly employed by farmers, and 303 workers are employed by farm labor contractors. In the study, four workers did not answer the employment type question. Table 1 shows the summary statistics for worker information and data for each of the two pay arrangements.

**Table 1:** Summary statistics of worker effort, heat exposure and worker demographic characteristics for 575 workers.

		All workers		Workers paid by piece rate		Workers paid by hourly rate		Test
Numerical Variables	unit	Number of obs.	mean (s.d.)	Number of obs.	Mean (s.d.)	Number of obs.	Mean (s.d.)	t-score statistic ^e^
hourly METs ^a^	kcal/h	6650	1.8	1289	2.0	5361	1.7	11.5*
			(0.6)		(0.7)		(0.6)	
Hourly WBGT ^b^	˚C	6650	24.2	1289	22.7	5361	24.5	12.9*
			(4.9)		(4.6) ^e^		(4.9)	
Shift length	H:M	575	8:4	123	7:2	452	9:1	13.1*
			(1:3)		(1:1)		(1:2)	
Age	Year	575	38.6	123	35.4	452	39.4	3.8*
			(12.0)		(11.0)		(12.1)	
Body Mass Index ^c^	Index	575	29.1	123	27.8	452	29.4	3.9*
			(4.7)		(3.9)		(4.8)	
Categorical Variables		All workers	Workers paid by Piece Rate	Workers paid by hourly rate			Chi-squared statistic ^f^
		Number of obs. Share %		Number of obs. Share %		Number of obs. Share %		
All				123		452		
				21.4%		78.6%		
Gender								1.0
Male		378		86		292		
		65.7%		15.0%		50.8%		
Female		197		37		160		
		34.3%		6.4%		26.1%		
Hire Type ^d^								9.9*
Direct Hire		268		41		227		
		46.6%		7.1%		39.5%		
Contractor		303		80		223		
		52.7%		13.9%		38.8%		

Notes

^a^ The range of the average metabolic equivalents (METs) from 1 to 2 is similar to the range of energy expenditure from sedentary activities such as sitting [20].

^b^ The calculation of hourly Wet Bulb Globe Temperature (WBGT) could be found in Section 3.

^c^ Based on World Health Organization BMI classification, an individual is considered 1) normal if 18.5≤BMI<25, 2) overweight if 25≤BMI<30, 3) obese if BMI>30.

^d^ Four workers have no hire type information, with two paid by piece rate and two workers paid by hourly wage rate.

^e^ T-score statistic greater than 1.96 implies statistical significance in different mean values.

^f^ Chi-square statistic greater than 3.84 implies correlation between pay type and gender (male or female) or hire type (contractor employee or direct hire).

* Reject the null hypothesis with p-value less than 0.05.

The average hours of work per day is 8 hours and 42 minutes for a single shift, similar to findings in Mitchell et al. [23]. During the shift, the average of hourly worker effort measured in metabolic equivalents of tasks (METs) is about 1.79 kcal per hour. On average, workers start their shifts with 0.75 METs, with slow increase in METs until noon when they reach an average of 2 kcal per hour. In the afternoon, workers decrease their effort gradually until the end of the shift. About 47% of the 6650 worker-hours have METs within the range from 1 to 2, which is similar to the range of energy expenditure from sedentary activities such as sitting [26]. About 42% of the 6650 worker-hours have METs within the range from 2 to 3, which is similar to the range of energy expenditure from light activities such as walking [26]. The other 11% of the worker-hours have the METs less than 1, which is the range of energy expenditure when the workers are resting. The energy expenditures of workers in these data are likely to be underestimated because the waist-worn devices that measured the workers’ effort are unable to capture the upper arm movement, which are significant in many farm tasks performed. We controlled for tasks in our regression model, and any undermeasurement of activity due to the device location occurs for all workers and is independent of workers’ pay arrangements and their heat exposure. Therefore, these data can still provide a source for unbiased parameter estimates of the effort of worker in response to pay arrangements and heat exposure.

The average hourly heat exposure measured in WBGT is 24.18˚C. During the shift, the average hourly heat exposure in the early morning is around 15 to 20˚C, then slow increases over the time of the day, with the maximum in the early afternoon (2:00 to 3:00 pm), which reaches close to 30˚C. The heat exposure decreases in the late afternoon, with an average heat exposure around 24˚C, which is still much higher than the heat exposure in the morning. About 48% of the 6650 worker-hours face heat exposure within the range from 15 to 25˚C. About 49% of the 6650 worker-hours have heat exposure within the range from 25 to 35˚C. When the WBGT is higher than 26˚C, working or exercising for over 45 minutes in direct sunlight have an increased likelihood of heat stress leading to heat-related illness symptoms.

The second and third column in the top panel of Table 1 show summary statistics for the four numerical variables, effort, heat exposure, age and BMI by pay arrangements. Workers paid by piece-rate arrangements have on average higher effort (in metabolic equivalents) but a shorter shift length than workers paid by hour wages. The average hourly WBGT during the shift for piece rate workers is lower than the average hourly WBGT for hourly paid workers. Workers paid by piece-rate arrangements are younger and have lower BMI than hourly paid workers. These differences in mean values across workers are statistically significant as shown in the column labeled “t-score,” which provides the test statistics for the test of differences in means.

The bottom panel of Table 1 shows summary statistics for categorical variables by pay arrangements. The female and male worker ratios within workers paid by piece-rate arrangements and workers paid by hourly wages are similar, with about twice the number of male workers than female workers. We use the chi-square test to estimate whether the female and male ratio within each pay arrangement is statistically different from the sample female and male ratio (197 female to 378 male workers ratio). The chi-square statistic is 0.98, implying that we do not reject the test null hypothesis at 0.05 significance level that the ratio of the female and male workers between hourly paid workers and piece rate workers are the same as the female to male ratio in the dataset.

The ratio of direct farm employees to those employed by contractors is higher among workers paid by hourly wages than workers paid by piece-rate arrangements. Among workers paid by hourly wages, there are about the same number of workers employed directly by the farmers and workers employed by farm labor contractors. Among the workers paid by piece-rate arrangements, the ratio of direct hires to contractor hire is about 0.5. The chi-square statistic is 9.85 implying that the ratio of the direct farm employee and contractor employee workers between workers paid by hourly wages is different from the ratio among workers paid by piece-rate arrangements at significance level 0.05.

Table 2 provides a detailed description of the agricultural tasks, showing the number of workers in each task overall and by pay arrangements. Among the 575 workers, about one third of workers (170 workers) worked on harvesting tasks, with about 110 workers harvesting tree fruits, and 60 workers harvesting other crops. Another 20% of workers (111 workers) work on thinning and pruning tasks, with about 74 workers thinning and pruning tree crops, and 37 workers thinning and pruning other crops. About 17% of workers work on harvest-related tasks, such as carrying (9 workers), sorting (48 workers), packing (23 workers) and supervising (18 workers). The rest of the workers work on other farm works such as irrigation (73 workers), hoeing and raking (29 workers), shoveling (9 workers) and nursery related tasks (12 workers).

**Table 2:** Task descriptions and number of workers by pay arrangement.

Tasks	All		Piece rate		Hourly wage		Activities
All	575	%	123	%	452	%	
Multi-task	72	12.5	6	4.9	66	14.6	No single task, a combination of the other 12 tasks.
Irrigation	73	12.7	0	0.0	73	16.2	Carrying/lifting and setting irrigation pipes. Opening/adjusting water valves. Reeling in irrigation lines. Movements include walking, bending, squatting. Often move equipment between fields using an off-road vehicle. Crops: none, tomato, grape, walnut, corn, carrot, garlic, variety
Ground pruner	37	6.4	1	0.8	36	8.0	Removing, trimming or thinning plants. Movements include walking, standing, bending, repetitive hand motion with shears or pruning tools. Crops: melon, basil, cotton, strawberry, tomato
Tree pruner	74	0.0	20	0.0	54	0.0	Removing, thinning, training branches, immature fruit, vegetation. Movements include walking, lifting tools above waist, repetitive hand motion with shears or pruning tools. Crops: plum, apricot, peach, grape, pistachio, almond
Harvest low	60	12.9	21	16.3	39	11.9	Hand harvesting into a container held on the body or carried. Movements include walking, standing, reaching, bending. Crops: raspberry, melon, cherry tomato, tomato, walnut, basil, flower, cucumber, pepper
Harvest high	110	0.0	68	0.0	42	0.0	Hand harvesting, usually stone fruit. Movements include walking, carrying ladder, climbing ladder, reaching, placing crop into container and carrying to a bin. Crops: plum, peach, olive
Hoeing and raking	29	10.4	1	17.1	28	8.6	Using a hoe or rake to weed and remove plants. Movements include walking, stooping and dragging matter with tools. Crops: none, walnuts
Shoveling	9	19.1	0	55.3	9	9.3	Using shovels to lift, dig, or move bulk material, such as soil, nuts or hulls. Movements include walking, standing, shoveling. Crop: fallow, tomato, almond
Sorting	48	5.0	1	0.8	48	6.2	Separating harvested crops by grade/size. Mostly standing, with repeated arm and hand movement. Crops: peach, sweet potatoes, pepper, cherry tomato, tomato
Carrying	9	1.6	1	0.0	8	2.0	Moving ready-packed crops or other materials. Loading and unloading of materials. Movements include bending, walking, lifting bulky materials. Crops: melon
Packing	23	8.3	3	0.8	20	10.6	Packing crops into boxes or other shipping material. Loading and unloading boxes. Movements include sitting, standing, forming boxes, packing and moving filled boxes, some on a harvester. Crops: melons, raspberry, tomatoes, variety
Supervisor or driver	18	1.6	1	0.8	17	1.8	Supervising and other work not directly on crops. Movement includes driving open cab vehicles and tractors, walking, standing, communicating, assisting, supervision. Crop: fallow, melon, peach, tomato, pistachio, almond
Nursery	12	4.0	0	2.4	12	4.4	Plant and maintain vegetation not grown as crops. Including athletic fields, lawns, trees, shrubs, flowerbeds and other landscaping elements. Movements include driving open-air vehicles, mowing, pruning, planting, weeding.

The majority of workers paid by piece-rate arrangements (109 out of 123 workers) worked on tree pruning/thinning and harvesting-low (no reaching up) and harvesting-high (which required reaching up and likely included work from ladders). In comparison, workers paid by hourly wages worked on tasks such as irrigation, raking and hoeing, sorting and packing, and multiple tasks in one shift. One reason that piece rate was seldom used for such tasks may be that it is difficult to consistently measure the unit of output for these tasks.

Table 3 shows the calendar month of tasks described in Table 2. The time schedule of tasks and corresponding crops is highly correlated to the months of the year. Tasks such as hand picking, sorting and packing, mostly occurred during July, August and September which are the hottest months of the year and also the harvest seasons for many fruits and vegetables in California. Tasks that are not directly related to any crops such as irrigation, hoeing and raking are more evenly distributed across months.

**Table 3:** The distribution of workers across tasks monthly.

Tasks	June		July		August		September		October	
**All workers**	67	%	202	%	137	%	154	%	15	%
**Multi-task**	12	17.9	20	9.9	15	10.9	25	16.2	0	0
**Irrigation**	28	41.8	3	1.5	8	5.8	34^b^	22.1	0	0
**Ground pruner**	1	1.5	33	16.3	0	0	3	1.9	0	0
**Tree pruner**	1	1.5	20	9.9	46	33.6	7	4.5	0	0
**Harvest low**	8	11.9	26	12.9	1^a^	0.7	25	16.2	0	0
**Harvest high**	0	0	59	29.2	32	23.4	6	3.9	13	86.7
**Hoe & Rake**	5	7.5	3	1.5	12	8.8	9	5.8	0	0
**Shoveling**	0	0	0	0	2	1.5	7	4.5	0	0
**Sorting**	0	0	13	6.4	12	8.8	24	15.6	0	0
**Carrying**	2	3.0	4	2.0	1	0.7	0	0	2	13.3
**Packing**	9	13.4	14	6.9	0	0	0	0	0	0
**Supervisor or driver**	1	1.5	7	3.5	8	5.8	2	1.3	0	0
**Nursery**	0	0	0	0	0	0	12	7.8	0	0

Notes

^a^ The data have only one worker working on harvesting low in August. This worker was surveyed with 6 other workers at the same facility and on the same day who were working on tasks such as irrigation, shoveling, sorting and operating more than one task.

^b^ The survey includes facilities located in the Imperial Valley. The growing season in Imperial Valley lasts from October to June, with the seeding and growing with irrigation in September.

Table 4 shows the shift starting hour and ending hour overall and for workers paid by the two pay arrangements. We observe that the starting time for workers paid by different pay arrangements are similar, with the majority workers starting between 5 am to 7 am. The majority of workers (107 out of 123 workers) paid by piece rate finished their shift by 3 pm, while more than half of the workers paid by hourly wage worked past 3 pm.

**Table 4:** Shift starting and ending hours for workers paid by piece rate and hourly rate.

	(1)	(2)	(3)	Equal average start/end hour across (2) and (3)
	All Workers	Workers paid by piece rate	Workers paid by hourly wage	t-stats
Number of workers	575	123	452	
Average start hour (H:M:S)	06:04:35	06:08:26	06:03:32	1.48 (<1.65)
4:00 AM—5:00 AM	9	0	9	
5:00 AM—6:00 AM	261	62	199	
6:00 AM—7:00 AM	261	50	211	
7:00 AM—8:00 AM	44	11	33	
Average end hour (H:M:S)	14:46:33	13:30:44	15:07:10	11.46 (> 1.65)
11:00 AM—12:00 AM	15	8	7	
12:00 AM—1:00 PM	67	46	21	
1:00 PM—2:00 PM	76	22	54	
2:00 PM—3:00 PM	142	31	111	
3:00 PM—4:00 PM	141	9	132	
4:00 PM—5:00 PM	86	4	82	
5:00 PM—6:00 PM	46	3	43	
6:00 PM—7:00 PM	2	0	2	

Notes: The shift start time is highly dependent on when the sun comes up, hence we observe the earliest start time in June.

### 4.2 Impact of piece-rate arrangements on effort: Without propensity score matching

The estimated impacts of piece-rate pay arrangements on workers’ effort show that the workers are exerting more effort when the WBGT is low, but less effort when the WBGT is high than workers paid by hourly wage.

Table 5 shows the estimated impacts of piece rate and WBGT on worker effort for a full sample with 571 workers. Four out of the 575 workers were excluded from the regression due to lack of data on whether they are employed directly by the farm or by a farm labor contractor. The results in Column 1 are based on model specifications excluding the heat exposure variables. The Column 1 results show that workers paid by piece-rate arrangements have 0.046 METs more per hour on average than workers paid by hourly wages, which is about 4% of the average metabolic rates a worker has per hour in our study shift. The estimated effect of piece-rate arrangements is not statistically significant.

**Table 5:** The impact of piece-rate arrangements and heat exposure (measured in WBGT) on workers’ effort (units in 0.01 Metabolic Equivalents, METs) based on regression results using a full sample.

	Dependent variable: Metabolic rate		
	(1)	(2)	(3)
WBGT		7.98	7.64
		(1.37)	(1.56)
WBGT^2		-0.20	-0.19
		(0.026)	(0.030)
Piece rate	4.60		-63.18
	(3.09)		(38.33)
Piece rate: WBGT			8.37
			(3.44)
Piece rate: WBGT^2			-0.23
			(0.078)
Time of day	0.55	0.59	0.60
	(0.017)	(0.024)	(0.024)
Time of day ^2	-0.00028	-0.00029	-0.00029
	(0.0000086)	(0.0000105)	(0.000011)
Female	-13.75	-13.30	-13.22
	(2.94)	(2.94)	(2.94)
BMI	-0.22	-0.25	-0.24
	(0.21)	(0.22)	(0.22)
Age	-0.25	-0.22	-0.21
	(0.096)	(0.095)	(0.096)
Hired by contractors	5.69	5.82	5.60
	(2.65)	(2.60)	(2.63)
Task and Month fixed effects	Yes	Yes	Yes
R-square (without fixed effects) (%)	21.1	22.9	23.4
R-square (with fixed effects) (%)	29.1	30.7	31.1
N. of workers	571	571	571
N. of worker hour	6603	6603	6603

Notes: Other covariates include nonlinear hours of day, task types, age, BMI, hire type, gender and month of the year. The standard error is worker-ID cluster-robust standard error. Values in parentheses are standard errors. Column 1 represents results from regression model without no heat exposure variables, while including the pay arrangement variable, and all fixed effects. Column 2 represents results from regression model without the pay arrangement variable but including heat exposure variables and fixed effects. Column 3 includes both heat exposure variables, pay arrangement variables, their interactions, and all fixed effects.

The results in Column 2 are based on model specifications excluding pay arrangement but including the heat exposure variables. The Column 2 results show that workers increase their effort when the heat exposure is low and decrease their effort when the heat exposure is high. The marginal response of effort to heat exposure for an average worker is

$$\frac{\partial mets}{\partial wbgt}=7.98-0.4wbgt.$$

In Column 3 where both the heat exposure variables and pay arrangements variables are included in the specification (Eq 1), the results show that the workers paid by piece-rate arrangements have statistically significant lower effort than workers who were paid by hourly wage. The estimated effect of piece rate on workers’ effort is

$$\frac{\partial mets}{\partial PieceRatePay}=-63.18+8.37wbgt-0.23wbgt^{2}$$

If measured at the mean WBGT (24.18˚C), workers paid by piece-rate arrangements expend 0.047 METs more per hour than workers paid by hourly wages. The point estimate of the effect of piece-rate arrangements on workers’ effort is very similar to the 0.046 METs estimated in Column 1 when the model omits the heat exposure variables.

In the following, we interpret the results in Column 3 where all variables are included. For workers paid by hourly wages, the marginal response of effort to heat exposure is:

$$\frac{\partial met_{hour}}{\partial wbgt}=7.64-0.38\;wbgt$$ (1A)

For workers paid by piece-rate arrangements, the marginal response of effort to heat exposure is

$$\frac{\partial met_{piece}}{\partial wbgt}=(7.64+8.37)-2\times (0.19+0.23)\;wbgt$$ (1B)

For comparison, we plot the marginal response of the energy expenditure with respect to the heat exposure levels for workers paid by different pay arrangements.

**Fig 1:**
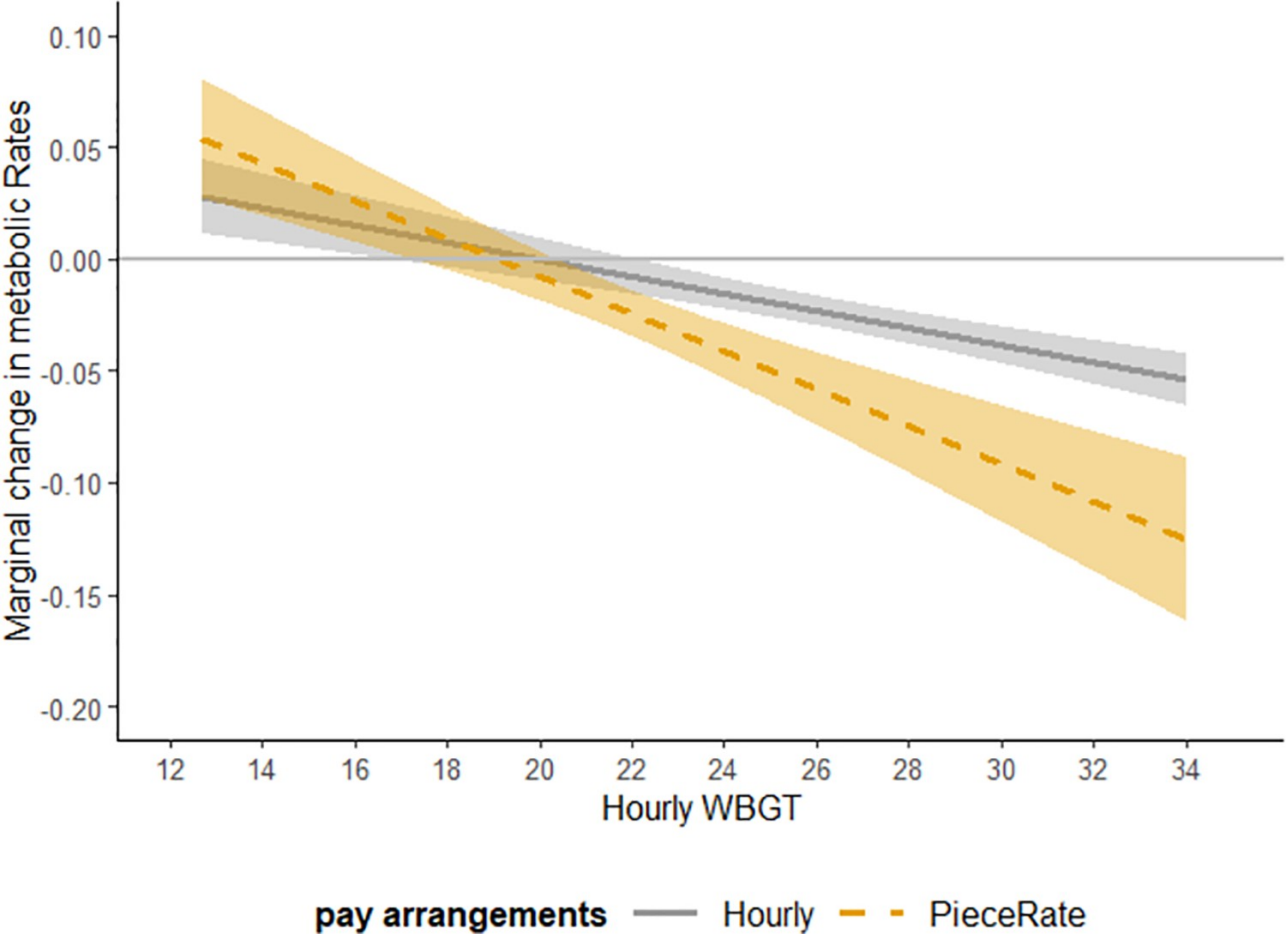
Marginal response in effort with respect to WBGT for workers paid by different pay arrangements based on regression coefficients from Table 5 Column 3. Note: The solid gray line shows the marginal change in workers’ effort for whom is paid by hourly wages when there is one degree increase in heat exposure. The dotted yellow line shows the marginal change in workers’ effort for whom is paid by piece rates when there is one degree increase in heat exposure. The gray bound around each line shows he 95% confidence intervals.

Fig 1 shows the marginal response of effort with respect to heat exposure measured in WBGT for workers paid by different arrangements. The solid line is the marginal response of effect for workers paid by hourly arrangements based on Eq (1A). The dashed line is the marginal response of effect for workers paid by piece-rate arrangements based on Eq (1B). The ribbons around the lines are the 95% confidence intervals. When the heat exposure level is low, an increase in heat causes workers to increase their effort. When the heat exposure is high, exceeding the threshold around 19 to 20˚C, an increase in heat causes workers to decrease their effort.

Fig 1 also illustrates how the response to heat exposure differs by pay arrangements. Workers paid by piece-rate arrangements show larger effects of heat exposure on their effort compared to workers paid by hourly arrangements (the slope of the marginal response is steeper for workers paid by piece-rate payments). When the heat exposure level is low, an increase in heat causes workers paid by piece-rate arrangements to increase effort more than workers paid by hourly wage arrangements. When the heat exposure is high, exceeding the threshold of 19 to 20˚C, an increase in heat causes workers paid by piece-rate arrangements decrease effort more workers paid by hourly wage arrangements.

In order to compare the worker effort under different heat exposure levels between piece rate and hourly rate, we estimate the difference in worker effort as:

$$\beta_{1}+\beta_{4}wbgt_{ih}+\beta_{5}wbgt_{ih}^{2}.$$

Using the coefficient estimate from Column 3 from Table 5, we have:

$$\Delta ={\hat{mets}}_{piece}-{\hat{mets}}_{hour}=-63.18+8.37\;wbgt-0.23\;wbgt^{2}.$$

**Fig 2:**
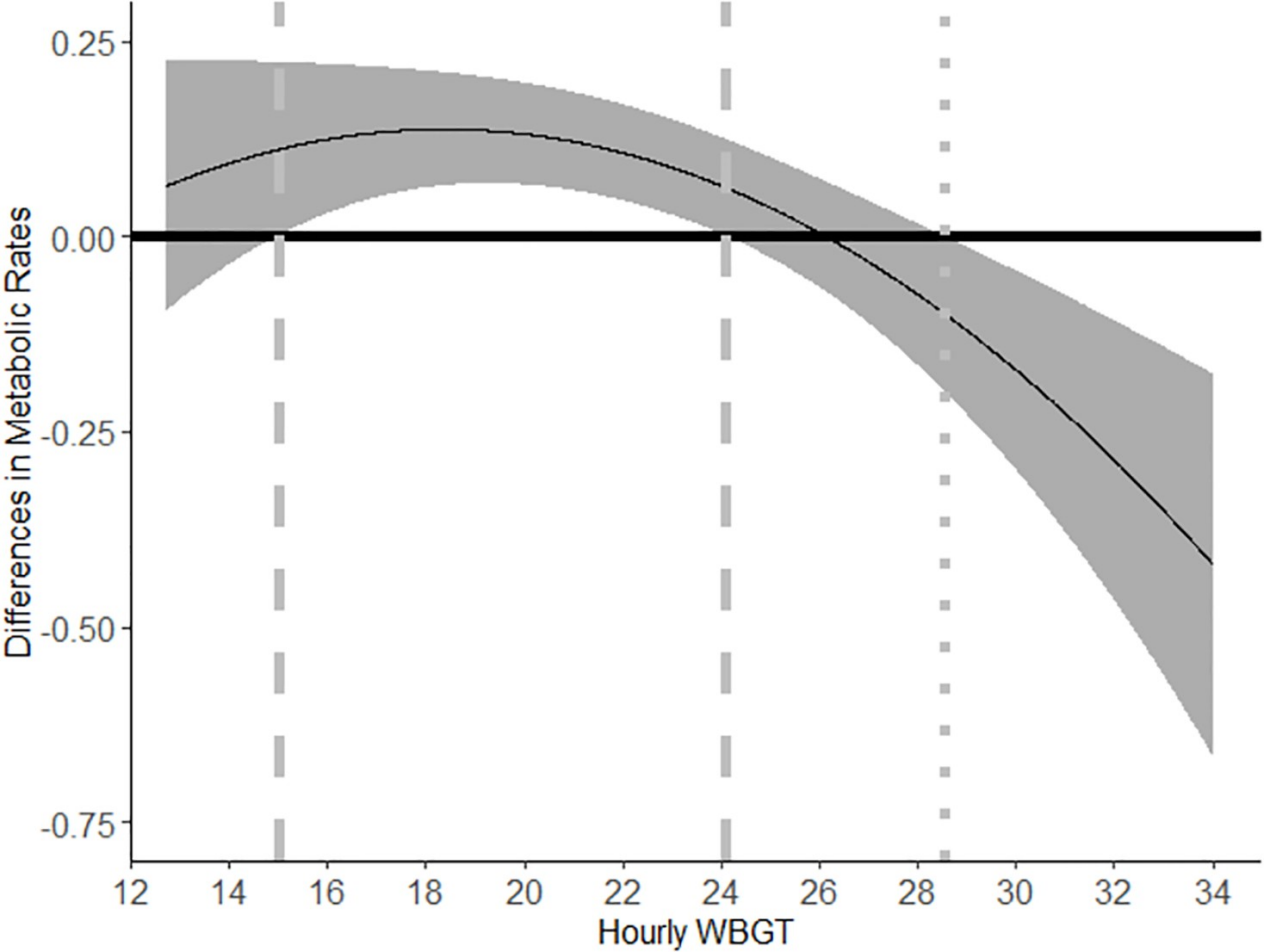
Differences in effort between workers paid by piece-rate arrangements and hourly wages in response to WBGT based on regression coefficients from Table 5 Column 3. Note: The solid line curve shows the differences in worker effort between workers paid by different schemes. The gray area shows the 95% confidence interval of the metabolic differences. The dot-dashed lines show the cut-off points of hourly WBGT where the estimated 95% confidence interval of the differences in metabolic rates between workers paid by piece rate and hourly rate is above zero. The dashed lines show the cut-off points of hourly WBGT where the estimated 95% confidence interval of the differences in metabolic rates between workers paid by piece rate and hourly rate is below zero.

Fig 2 shows the estimated differences in worker effort in response to heat exposure when paid by different pay arrangements using the full sample estimate in Column 3 of Table 5. The quadratic curve represents the point estimate of the difference between workers’ effort when paid by piece rate and hourly rate conditional on WBGT. The gray band represents the confidence interval estimated using the cluster robust standard error from Table 5. The dot-dashed lines show the cut-off points of hourly WBGT where the estimated 95% confidence interval of the differences in metabolic rates between workers paid by piece rate and hourly rate is above zero, i.e., workers exert significantly more effort under piece rate than workers paid by hourly rate. The dashed lines show the cut-off points of hourly WBGT where the estimated 95% confidence interval of the differences in metabolic rates between workers paid by piece rate and hourly rate is below zero, i.e., workers exert significantly less effort under piece-rate arrangements than workers paid by hourly wages. Based on Fig 2, workers exert more effort when the WBGT ranges from 15˚C to 24.11˚C. When WBGT ranges from 24.11˚C to 28.54˚C, we do not find a statistically significant impact of piece-rate arrangements on worker effort compared to hourly paid workers. When WBGT exceeds 28.54 ˚C, we find workers paid by piece-rate arrangements exert less effort than hourly paid workers.

### 4.3 Impact of piece-rate arrangements on effort using 216 workers: with propensity score matching

To use propensity score matching, we first selected workers based on their tasks. We include all workers who perform the tree pruning or thinning task, or the harvesting task. Then, using the propensity score matching technique, we selected 216 workers of those workers who were pre-selected based on their tasks. More details regarding the results for the first matching step are in the S1 appendix.

Using propensity score matching, Table 6 shows the results of estimating the main regression, Eq (1) using the matched 216 workers. Note that there were 109 workers paid by piece rate workers, with one of them having missing information of contractor type. We successfully matched all 108 workers paid by piece-rate arrangements with complete information with workers paid by hourly wage rate (resulting in 216 workers in the sample). In Column 1, workers paid by piece rate have statistically significant higher average effort (0.0698 METs/hour) than workers paid by hourly wages, conditional on task types, hiring type and workers characteristics. In Column 2, workers have nonlinear responses to heat exposure, with an inverse U-shaped functional form. The marginal response of effort level to heat exposure is

$$\frac{\partial mets}{\partial wbgt}=4.41-0.3\;wbgt.$$

**Table 6:** The impact of piece rate and heat exposure (measured in WBGT) on workers’ effort (units in 0.01 METs) based on regression results using a matched sample (Values in parentheses are standard errors).

	Dependent variable: Metabolic rate		
	(1)	(2)	(3)
WBGT		4.41	-1.94
		(2.54)	(3.64)
WBGT^2		-0.15	0.00012
		(0.051)	(0.072)
Piece rate	6.98		-142.31
	(3.52)		(49.43)
Piece rate: WBGT			15.64
			(4.41)
Piece rate: WBGT^2			-0.38
			(0.097)
Time of day	0.73	0.78	0.81
	(0.033)	(0.045)	(0.049)
Time of day^2	-0.00038	-0.00039	-0.00040
	(0.000018)	(0.000021)	(0.000023)
Female	-13.97	-12.18	-12.25
	(3.50)	(3.51)	(3.48)
BMI	-0.38	-0.38	-0.35
	(0.38)	(0.37)	(0.38)
Age	-0.21	-0.24	-0.19
	(0.17)	(0.16)	(0.16)
Hired by contractors	0.95	0.0102	-0.51
	(4.08)	(4.28)	(4.27)
Task and Month fixed effects	Yes	Yes	Yes
R-square (without fixed effects) (%)	27.2	28.8	29.2
R-square (with fixed effects) (%)	30.2	31.0	32.2
N. of workers	216	216	216
N. of worker hour	2341	2341	2341

Notes: Other covariates include nonlinear hours of day, task types, age, BMI, hire type, gender and month of the year. The standard error is worker-ID cluster-robust standard error. Column 1 represents results from regression model without no heat exposure variables, while including the pay arrangement variable, and all fixed effects. Column 2 represents results from the regression model without the pay arrangement variable but including heat exposure variables and fixed effects. Column 3 includes both heat exposure variables, pay arrangement variables, their interactions, and all fixed effects.

In Column 3, results show that the effect of piece-rate arrangements on workers’ effort is

$$\frac{\partial mets}{\partial PieceRatePay}=-142.31+15.64\;wbgt-0.38\;wbgt^{2}$$

The coefficient in front of piece-rate pay arrangement is negative, i.e., -142.31kcal/h. However, the effect of piece rate on energy expenditure is positive for a large relevant range below the mean heat exposure, because of the sign and magnitude of the interaction term between piece rate and WBGT, within the WBGT ranging from 14 to 28˚C. With the mean heat exposure of 23.32˚C, the workers paid by piece-rate arrangements spent 0.15 METs effort more than did workers paid by hourly wage. The piece-rate effect estimated with all interaction variables included as shown in Column 3 is about twice as larger as the effect estimated in Column 1 where no WBGT variables are included in the regression. One interpretation is that when no WBGT variables are included in Column 1, it causes a biased estimate due to the omitted heat exposure variable in the error term, which is correlated with piece rate. Without including the WBGT variables, the results in Column 1 are underestimating the piece-rate pay arrangement on workers’ effort because the piece-rate pay arrangement captures some effects of heat exposure. At the mean heat exposure, increasing heat exposure has a negative effect on workers’ effort. If piece-rate arrangements are positively correlated with heat exposure, then the specification in Column 1 leads to underestimate the impact of piece-rate pay arrangement.

The marginal response of effort level to heat exposure is

$$\frac{\partial me{ts}_{hour}}{\partial wbgt}=-1.94+0.00024\;wbgt.$$ (2A)

With the mean WBGT at 23.97˚C, a one degree increase in the mean WBGT decreases the energy expenditure measured in METs by 1.93 kcal/h, which is not statistically significant from 0. The marginal response of effort level to heat exposure for workers paid by piece-rate arrangements is

$$\frac{\partial me{ts}_{piece}}{\partial wbgt}=(15.64-1.94)-2\times (0.38-0.00012)wbgt.$$ (2B)

**Fig 3:**
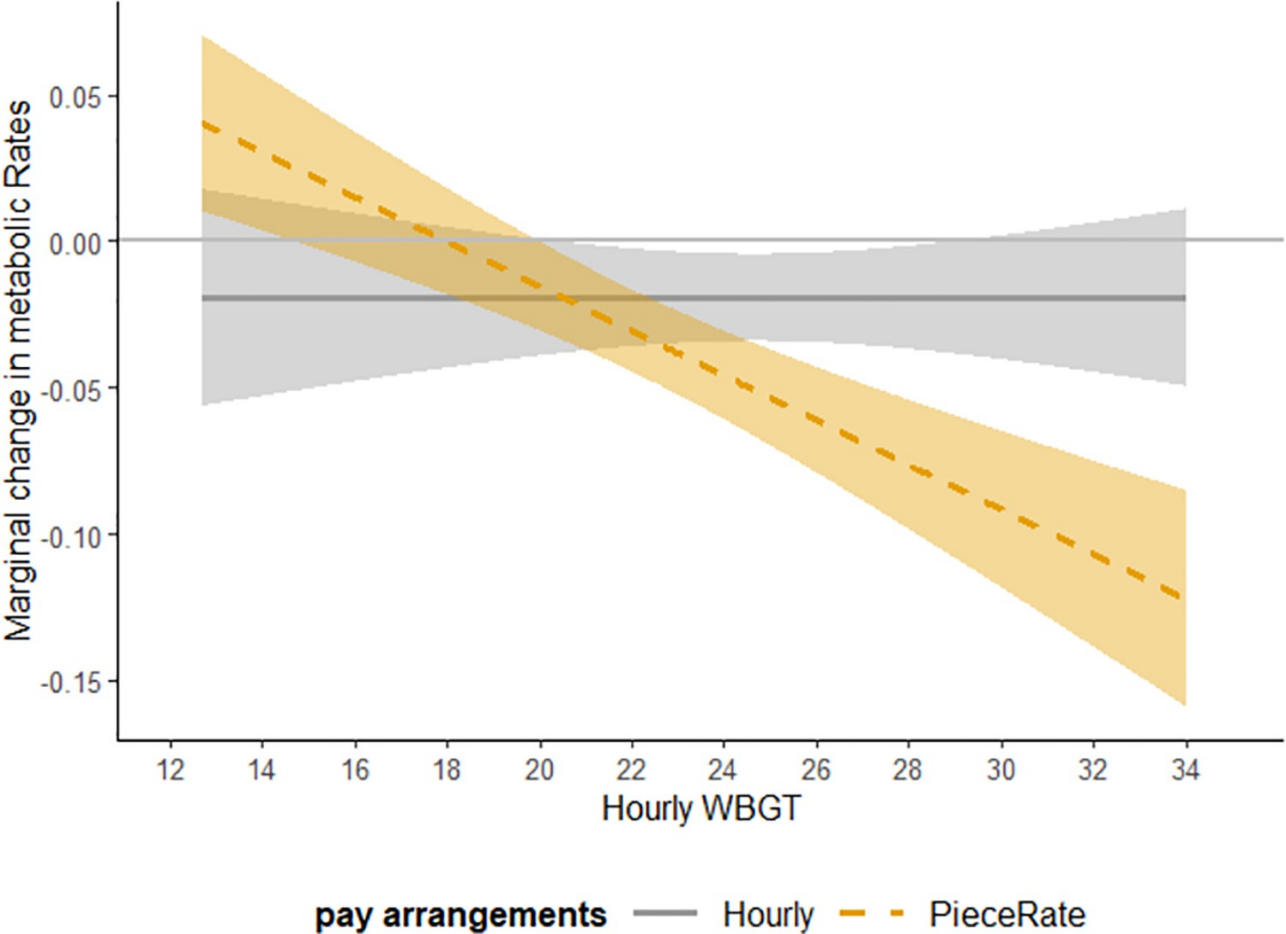
Marginal response in effort with respect to WBGT for workers paid by different pay arrangements based on regression coefficients from Table 6 Column 3. Note: The solid gray line shows the marginal change in workers’ effort for whom is paid by hourly wages when there is one degree increase in heat exposure. The dotted line shows the marginal change in workers’ effort for whom is paid by piece rates when there is one degree increase in heat exposure. The gray bound around each line shows the 95% confidence intervals.

Fig 3 shows the marginal response of effort with respect to heat exposure levels measured in WBGT for workers paid by different pay arrangements. The solid line is the marginal response of effect for workers paid by hourly pay arrangement based on Eq (2A). The dashed line is the marginal response of effect for workers paid by piece-rate pay arrangement based on Eq (2B). The ribbon around the lines are the 95% confidence intervals. Fig 3 shows the workers paid by hourly wage arrangements have a constant and close to zero response in effort when the heat exposure changes, while workers paid by piece-rate arrangements have a much larger marginal response of effort across different heat exposure levels. When the heat exposure level is low, workers paid by piece-rate arrangements increase their effort. When the heat exposure is high and exceeds a threshold (around 19 to 20˚C), workers decrease their effort.

With the mean WBGT at 22.52˚C, a one degree increase in the mean WBGT decreases the energy expenditure measured in METs by 3.49 kcal/h, which is statistically significantly different from 0. To compare the worker effort under different heat exposure between piece rate and hourly rate, we calculate the difference in workers’ effort level as

$$\beta_{1}+\beta_{4}WBGT_{ih}+\beta_{5}WBGT_{ih}^{2}.$$

Using the coefficient estimate from Column 3, we have:

$$\Delta ={\hat{mets}}_{piece}-{\hat{mets}}_{hour}=-142.31+15.64\;WBGT-0.38\;WBGT^{2}.$$

**Fig 4:**
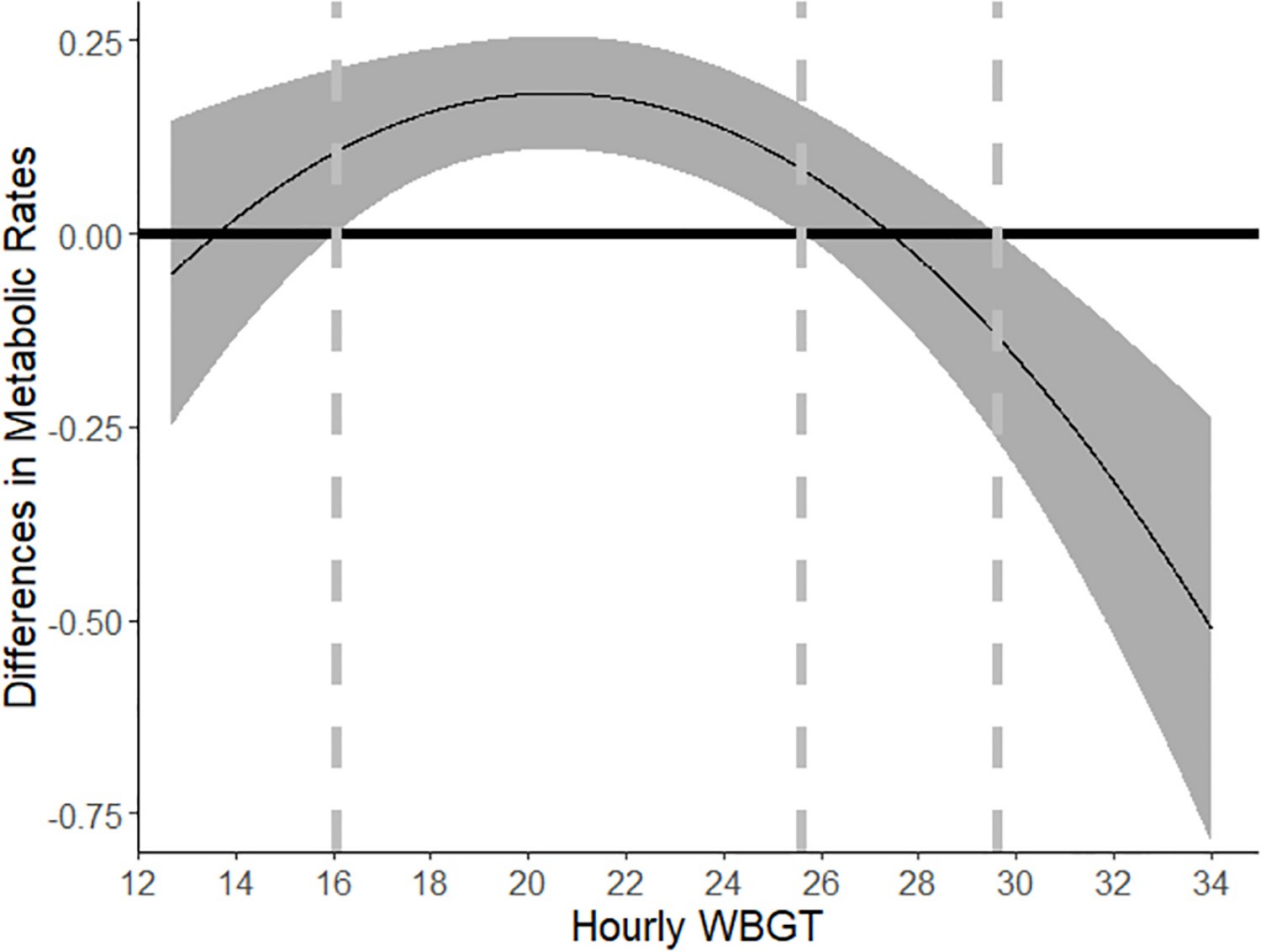
Differences in effort between workers paid by piece rate and hourly wage rate in response to WBGT based on regression coefficients from Table 6. Note: The dot-dashed lines are the cut-off points of hourly WBGT where the estimated 95% confidence interval of the differences in metabolic rates between workers paid by piece rate and hourly rate is above zero. The dashed lines are the cut-off points of hourly WBGT where the estimated 95% confidence interval of the differences in metabolic rates between workers paid by piece rate and hourly rate is below zero.

Fig 4 shows the projected difference in effort in response to heat exposure when paid by different pay arrangements using the matched sub-sample estimate in Column 3 Table 6. Based on Fig 4, workers exert more effort when the WBGT ranges from 16.07˚C to 26.61˚C. When WBGT ranges from 25.61˚C to 29.62˚C, we do not find a statistically significant impact of piece rate on workers’ effort level compared to hourly paid workers. When the WBGT exceeds 29.62˚C, workers who are paid by piece-rate arrangements exert less effort than hourly workers.

Comparing Tables 5 and 6, and Figs 2 and 4, the main message is robust to the regression results using either a full sample or a matched sample. In summary, workers reduce their effort in the beginning when the heat exposure is moderate. The change in effort is likely due to workers’ planned effort over the course of full shift. For example, when workers expect heavy activity for a long period during the shift and high heat exposure during the latter half of the shift, they likely intentionally raise their effort when the heat exposure is low, so that they can lower effort during hot time and thereby have less time with severe heat exposure because they can get sufficient work done earlier. With more flexible shift hours per day than workers who are paid by hourly wages, workers who are paid by piece-rate arrangements have more flexibility to adjust their effort across the day.

The main difference between the results using a matched sample and a full sample is that the matched sample results in a larger estimate of the impact of piece-rate arrangements on workers’ effort. The results in Column 3 in Table 6 shows that the marginal effect of piece rate is 0.15 MET’s which is three times larger than the estimated marginal effect of piece rate measured in Table 5, Column 3. In addition, the workers’ response to heat exposure is

$$\frac{\partial mets_{piece}}{\partial wbgt}=13.7-0.75wbgt$$

based on Table 6 results, with -3.26 kcal/h (with standard error 5.7kcal/h) measured at the mean WBGT. In comparison, the marginal effect of heat exposure on the energy expenditure is

$$\frac{\partial mets_{piece}}{\partial wbgt}=16.01-0.84wbgt$$

from Table 5, with -3.05 kcal/h (with standard error 3.78kcal/h) measured at the mean WBGT. At the mean WBGT, the estimated effect of heat exposure on energy expenditure from full sample and matched samples are not statistically different from zero.

The differences in the coefficient estimate between Tables 5 and 6 are likely due to selection bias when using a full sample in Table 5. Recall that the matched sample utilized all 108 workers paid by piece-rate arrangements with complete information, matched with 108 workers paid by hourly wage rates. The only sample differences between Tables 5 and 6 are the unmatched workers who were paid by hourly wages. These workers tend to have very different tasks, i.e., irrigation, shoveling and raking, than tasks of workers paid by piece rate. Hence, dropping these workers paid by hourly wage rate who do tasks unsuited for piece rate helps provide unbiased estimates of the impact of piece-rate arrangements.

The results from a series of robustness checks reported in detail in the S1 Appendix are consistent with the main findings of the study reported in Tables 5 and 6 (See S1 Appendix). The robustness checks confirmed the findings that piece-rate arrangements increase worker effort early in the day but reduce worker effort when temperatures are higher later in the day.

## 5. Discussion

This section compares workers’ heat exposure (measured in WBGT) to Recommended Exposure Limits (RELs) suggested by National Institute for Occupational Safety and Health (NIOSH). We also offer limitations about our propensity score matching method and data. Finally, we discuss important implication of climate change for the importance of our results.

### 5.1 Heat exposure and recommended exposure limits

The study has been unable to directly measure whether workers experience “heat stress” during the sampled workdays. We find that piece-rate arrangements increase the workers’ effort when the heat exposure is moderate, but that workers reduce effort before the heat exposure becomes excessive. To evaluate whether workers experienced heat stress, we compare the Recommended Exposure Limits (RELs) of the National Institute for Occupational Safety and Health (NIOSH) to the actual hourly WBGT during the worker shifts in our sample. When the WBGT experienced by workers is above the RELs, there is an increased risk of heat stress [27]. Guidelines from NIOSH recommend that workers with higher metabolic activities work when there are lower WBGT in order to avoid heat strain and adverse health impact from heat exposure. The calculation of RELs as a function of hourly effort (in metabolic equivalents in Watts, METs) estimate is

REL=56.7–11.5log(mets).

The recommended exposure limits are for healthy workers who are physically fit for the activity required by their jobs. The workers are assumed to wear one-layer of conventional work clothing and work one-hour long periods without rest.

Given these definitions and methods, in this study, we do not observe exposures above the temperature established as the threshold for the presence of heat stress. The estimated WBGTs for the participating farm fields are below the Recommended Exposure Limits (REL), established by NIOSH [27], at the efforts we measured. We note that due to the nature of the study and the data collection process, it would not have been ethical to allow, or observe, actual heat stress among workers while the researchers were monitoring workers closely. We also note that the activity count per minute in our data likely underestimates the actual worker effort, which causes lower exposure estimate. As noted above, Mitchell et al [23] point out that the devices used to record activity count per minute are likely to underestimate energy expenditure for upper-body tasks, such as hand picking, pruning, thinning, and sorting. Thus, the RELs for these workers are overestimated. Therefore, heat stress is underestimated. Note, even with the concern of likely measurement error in workers’ effort, the parameter estimates in the Result Section remain unbiased because the suspected measurement errors are independent of heat exposure levels and pay arrangements.

The error in measuring level of effort is likely correlated to worker tasks. For example, for tasks that require mostly upper body movements, such as picking and pruning, the measurement error is likely to be large. In our regression models, we control for tasks both as explanatory variables and in the specification using full and matched samples. The measurement error in the error term is unlikely to be correlated to heat exposure or pay arrangements, hence the parameter estimates are unbiased.

### 5.2 Caveats related to our propensity score matching application

One of the concerns with propensity score matching (PSM) is “PSM paradox” [28]. The “PSM paradox” is the fact that propensity score matching may sometimes fail to match data as expected. However, we find that the magnitude of the “PSM paradox” impact in this study is small. First, as shown in Table 1, the 575 workers used to estimate the propensity score matching have large differences in gender distribution, BMI and age among workers paid by different pay arrangement. Second, the propensity score estimated for workers paid by different pay arrangements are very different (See S1 Appendix). The propensity scores for hourly paid workers are mostly below 0.5 and propensity scores for most piece-rate workers are above 0.5. Lastly, propensity score matching in this study appears to improve the distribution of worker demographics (See S1 Appendix), i.e., workers are more evenly distributed across different categorical variables, e.g., gender, compared to the differences among workers by pay arrangements in full sample (Table 1), and workers by pay arrangements in workers who were harvesting and thinning (See S1 Appendix). All the evidence indicates small problems with the propensity score matching method as applied here.

### 5.3 Data limitations

As with all empirical studies, our results are limited by data issues. First, the workers in this sample differ in some respects from all employed farm workers in California farms. It is likely that only farms that comply with California/OSHA regulations, in terms of worker environment standards, agreed to allow researchers to closely monitor the workers. Hence, while we observe that farm workers were able to stop working in the early afternoon when there is high heat exposure, it may not be the case for all farms in California. Second, we do not observe day to day repeated observations for workers, which means that we cannot estimate how workers respond to consecutive days of high heat exposure. Third, the dataset was collected in areas with relatively low humidity, with low heat exposure in the morning, and high heat exposure in the afternoon. Heat exposure is correlated with the observation that workers increase effort during periods of low exposure and decrease effort during periods of high heat exposure. It is not clear whether we would get similar results if the data were collected in a region where humidity is high, or when shifts start with a higher heat exposure and ends with lower heat exposure.

### 5.4 Implication of climate change

Due to climate change, farm workers will likely face higher heat exposure in the future. One way climate change will increase farm workers’ heat exposure is by increasing the number of days with extreme heat [2]. Daily extreme temperatures are projected to increase substantially in the contiguous United States by the Fourth National Climate Assessment (NCA4), with 20 to 30 more days per year with a maximum over 32°C in most areas by 2050.

Our results suggest that projected increases in heat exposure due to climate change will likely decreases farm worker effort. However, we show that workers paid by piece-rate and hourly wage arrangements respond to heat exposure differently. Given more work flexibility to respond to daily patterns, workers paid by piece-rate arrangements likely have lower effort than workers paid by hourly wage arrangements during periods of high heat exposure (WBGT greater than 29.6°C or air temperature greater than 31.2°C), and workers paid by piece-rate arrangements have higher effort when the heat exposure is moderate (with WBGT from 16.1°C to 26.7°C, or air temperature from 15.5°C to 26.7°C). Managing pay arrangements in response to changes in extreme heat exposure may help lower heat exposure in farm workers as climate changes in coming years.

## 6. Conclusion

It is vital to understand how farm workers respond to high heat exposure and how potential work and pay arrangements affect worker responses. Our results in this study indicate that workers respond to heat exposure nonlinearly, i.e., workers increase effort when experiencing low heat exposure (early as the day is getting warmer) but reduce effort when experiencing high and rising heat exposure. Furthermore, workers’ response to heat exposure differs across different pay arrangements. Piece-rate pay arrangement increases average worker effort compared to hourly wages. However, the impact of piece-rate arrangements on worker effort interacts with heat exposure. When workers face moderate heat exposure (with WBGT ranging from 16.07˚C to 26.61˚C), the piece-rate arrangements increase workers’ effort, but piece-rate pay decreases worker effort when workers face high heat exposure (with WBGT exceeding 29.62˚C).

## Supporting information

S1 Appendix (DOCX)
