# Supplementary material for: Compensation incentives and heat exposure affect farm worker effort
Source: PLoS One. 2021 Nov 2;16(11):e0259459. doi: 10.1371/journal.pone.0259459 (PMC8562852; doi:10.1371/journal.pone.0259459)
Supplement: S1 Appendix — (DOCX) [file pone.0259459.s001.docx]

**S1 Appendix**

# Heat exposure metric: Wet Bulb Globe Temperature (WBGT)

The WBGT is a temperature index measured by a certified meter using three different types of equipment: 1) the natural wet bulb temperature measured with a wetted thermometer exposed to the wind and heat radiation, 2) the black globe temperature measured inside a 150mm diameter black globe, 3) and the air temperature measured with a “normal” thermometer shaded from the direct heat radiation.( Occupational Safety and Health Administration, 2011). The WBGT could be calculated from the meter records as:

$$wbgt=0.7\times WetBulbTemp+0.2\times GlobeTemp+0.1\times AirTemp$$

In addition to meter measured WBGT, we utilize hourly meteorological data to fill in missing values of WBGT due to meter malfunction or any other causes. The meteorological data is recorded at nearby weather stations in the California Irrigation Management Information System (CIMIS), namely air temperature, humidity level, solar radiation and wind speed. There are several mathematical models applied to calculate WBGT using weather station data (Bernard and Pourmoghani, 1999; Tonouchi, Murayama and Ono, 2006; Lijegren et al, 2008; Gaspar and Quintela, 2009). We use the formula from Lijegren et al (2008) to calculate outdoor WBGT because the formula is shown to be the most accurate among all the models (Lemke and Kjellstrom, 2012).

We use the meter recorded WBGT as the primary resource and weather station WBGT as secondary resource measuring workers’ heat exposure in the field, in recognition of shortcomings that the nearby weather stations may misrepresent workers' environmental microclimates. Minute level WBGT is aggregated to hourly WBGT by taking the average of the WBGT measured from certified meters for each hour of the day. Any missing values are filled using calibrated WBGT calculated from weather station data. In the case when the meter malfunctioned during the study period and we only have weather station WBGT information, we fill in the missing WBGT using calibrated weather station WBGT. We calibrate WBGT by regressing the meter WBGT measured from certified meters over the WBGT computed from meteorological data. The linear regression model used to predict the meter WBGT is

$${wbgt}_{meter}=3.9˚C+0.905 {wbgt}_{wthstn}$$

which is based on the estimates by the author of the chapter.

# Work effort metric: metabolic equivalents (METs)

To compute the metabolic equivalents (METs) for each worker over the hours, workers wear an activity counting device at their waists. The device records workers’ movements such as walking and climbing ladders, and each small movement, such as steps, is counted as an activity (Crouter et al., 2006). The device provides time series activity counts during the period of workers wearing the device. However, the waist-worn device is unable to capture movements in the upper body (Mitchell et al., 2017), for example arm movements when picking fruits. More detailed discussion regarding the device and limitations could be found in Mitchell et al. (2017). The conversion from activity count to metabolic equivalents (METs) is adopted from Crouter et al. (2006). The correlation between activity count per minute and metabolic equivalents estimated by Crouter et al. (2006) used in our study is

$mets = 1 if activity count per minute < 50$,

$mets =2.48\cdot exp(0.000135\cdot activity) if activity count per minute >50$.

# Initial data analysis and tests of hypotheses

In this section, we show a preliminary analysis using simple t-tests to understand the differences between workers paid by piece rate arrangements and workers paid by hourly wages. The primary goal of the preliminary analysis is to help understand why the heat exposure measured in hourly WBGT is higher for workers paid by hourly wages than workers paid by piece rate arrangements, as shown in the article in Table 1. It is important to understand the potential causes of different heat exposure levels because we want to understand potential confounding factors that cause different heat exposure levels and hence different effort across workers paid by different pay arrangements. We test two hypotheses about why the workers paid by piece rate arrangements had lower hourly WBGT than workers paid by hourly wages.

*Hypothesis A: tasks paid by piece rate arrangements allow workers to perform tasks at different times of day compared to tasks typically performed by workers paid by hourly wages. Hence, workers paid by piece rate arrangements have shorter exposure to the peak heat period of the day.*

*Null Hypothesis: workers paid by piece rate arrangements work the same hours of the day as workers paid by hourly wages.*

Workers typically start their day early in the morning and end their shift in the afternoon, with a lunch break in between. Working a shorter time period during the peak heat period can reduce heat exposure, especially in the afternoon. We observe that workers paid by piece rate arrangements have lower heat exposure in the data. We want to determine whether workers paid by piece rate arrangement end their work earlier in the afternoon. We also want to check why the workers end their workday early. The following are the potential explanations: 1) workers paid by piece rate arrangements start the work day earlier than workers paid by hourly arrangements, such that their workday is done earlier, or 2) workers paid by piece rate do less work per day, 3) workers paid by piece rate arrangements work more efficiently or intensively and hence complete the same amount of work in fewer hours per day, and if they start no later than other workers, they can end the workday earlier. All three explanations can all apply at the same time, however, (2) and (3) are hard to distinguish because we cannot observe the direct work output of workers in the data.

In testing this hypothesis, we checked whether the piece rate workers start and finish at same time as hourly workers. First, we control the type of tasks and then compare the workers’ schedule by pay arrangements. We subset the workers who are operating pruning/thinning, harvest-low and harvest-high, because the majority of the workers paid by piece rate arrangements operate these four tasks as shown in the article in Table 2. Secondly, we use t-statistics to test whether the average starting time and shift ending time are different across workers paid by piece rate arrangements and hourly wages.

Table S3 shows the distribution of workers’ shift start and end time across all workers who perform pruning and harvesting tasks. We compute the average start hour and end hour for workers paid by piece rate arrangements and hourly wages, and then construct t-statistics to test the null hypotheses: shift start hour and end hour are the same for workers who are paid by piece rate arrangements and workers who are paid by hourly wages. We find that the difference in the time of the day when workers finish their shift are statistically significant at 5% significance level, workers paid by piece rate arrangements end their shift earlier. However, we do not find statistical significance at 5 % level in the differences in the time of the day when workers start their shift.

# Table S3. Shift starting and ending hours for workers operating tree pruning/thinning and harvesting.

|  | (1) | (2) | (3) | Equal average start/end hour across (2) and (3) |
| --- | --- | --- | --- | --- |
|  | Workers pruning and harvesting | Workers paid by piece rate | Workers paid by hourly wage | t-stats |
| Number of workers | 244 | 109 | 135 |  |
| Average start hour (H:M:S) | 06:12:17 | 06:10:11 | 06:13:59 | 0.89 ( < 1.97) |
| 4:00 AM - 5:00 AM | 2 | 0 | 2 |  |
| 5:00 AM - 6:00 AM | 98 | 52 | 46 |  |
| 6:00 AM - 7:00 AM | 118 | 46 | 72 |  |
| 7:00 AM - 8:00 AM | 26 | 11 | 15 |  |
| Average end hour (H:M:S) | 14:15:19 | 13:26:37 | 14:54:38 | 9.43 ( > 1.97) |
| 11:00 AM - 12:00 AM | 9 | 6 | 3 |  |
| 12:00 AM - 1:00 PM | 49 | 42 | 7 |  |
| 1:00 PM - 2:00 PM | 30 | 22 | 8 |  |
| 2:00 PM - 3:00 PM | 60 | 27 | 33 |  |
| 3:00 PM - 4:00 PM | 72 | 7 | 65 |  |
| 4:00 PM - 5:00 PM | 22 | 4 | 18 |  |
| 5:00 PM - 6:00 PM | 2 | 1 | 1 |  |

Note: The critical value is 1.97 for two-sided t-statistical test with 5% significance, 242 degrees of freedom.

*Hypothesis B: Workers paid by piece rate arrangements tend to work in cooler working environments, such that they have lower heat exposure. The cool working environments could be cooler months of the year, cooler regions, or simply cool days.*

*Null Hypothesis: Workers paid by piece rate arrangements work in a similar heat exposure environment.*

It is possible farm employers are more likely to provide piece rate arrangements when the working environment is cool, i.e., cooler months of the year, cooler regions, or simply cool days. To test this hypothesis, we control the hour of the day, and compute the heat exposure measured in Wet Bulb Globe Temperatures (WBGTs) across the two groups of workers. We analyzed data on hourly temperature and worker effort for 575 field workers from 31 farms located in Central and Imperial Valleys in California for 82 days during the summer of 2014 and 2015.

Table S4 shows the comparison of hourly WBGT across workers paid by different arrangements. Negative WBGT differences indicate that the workers paid by piece rate arrangements experience lower heat exposure than workers paid by hourly wages. We find the WBGT experienced by workers with difference pay arrangements are statistically significantly different. The workers paid by piece rate arrangements face statistically significantly lower WBGT than workers paid by hourly wages, except from 11:00 AM to 1:00 PM. For most hour to hour comparisons, we reject the null hypothesis that workers paid by different pay arrangements work in similar heat exposure environments, at the 5% significance level.

Table S4. Comparison of average WBGT experienced by workers paid by hourly and piece rate arrangements for all 575 workers (Values in parentheses are standard deviations).

| WBGT | All workers | piece rate workers | Hourly rate workers | WBGT difference | T-value for WBGT difference = 0 |
| --- | --- | --- | --- | --- | --- |
|  |  | (s.d.) | (s.d.) |  |  |
| 5:00 AM - 6:00 AM | 19.51 | 18.46 | 19.81 | -1.35 | 2.92 |
|  | (4.20) | (2.80) | (4.49) | (0.46) |  |
| 6:00 AM - 7:00 AM | 17.88 | 17.03 | 18.13 | -1.10 | 4.51 |
|  | (3.24) | (2.55) | (3.38) | (0.24) |  |
| 7:00 AM - 8:00 AM | 19.63 | 18.06 | 20.03 | -1.97 | 6.25 |
|  | (3.70) | (3.24) | (3.70) | (0.32) |  |
| 8:00 AM – 9:00 AM | 22.30 | 21.22 | 22.59 | -1.37 | 5.12 |
|  | (3.40) | (2.90) | (3.46) | (0.27) |  |
| 9:00 AM -10:00 AM | 23.78 | 22.79 | 24.09 | -1.30 | 5.25 |
|  | (3.23) | (2.65) | (3.33) | (0.25) |  |
| 10:00 AM -11:00 AM | 25.49 | 24.52 | 25.79 | -1.27 | 5.08 |
|  | (3.25) | (2.49) | (3.40) | (0.25) |  |
| 11:00 AM - 12:00 AM | 26.88 | 26.58 | 26.96 | -0.38 | 1.56 |
|  | (2.88) | (2.42) | (3.00) | (0.24) |  |
| 12:00 AM - 1:00 PM | 27.79 | 27.50 | 27.88 | -0.38 | 1.55 |
|  | (2.80) | (2.41) | (2.90) | (0.25) |  |
| 1:00 PM - 2:00 PM | 28.34 | 26.77 | 28.60 | -1.83 | 5.23 |
|  | (2.93) | (2.86) | (2.87) | (0.35) |  |
| 2:00 PM - 3:00 PM | 28.18 | 25.99 | 28.43 | -2.44 | 8.61 |
|  | (2.58) | (1.80) | (2.54) | (0.28) |  |
| 3:00 PM - 4:00 PM | 28.17 | 26.39 | 28.26 | -1.87 | 2.74 |
|  | (3.44) | (2.53) | (3.46) | (0.68) |  |
| 4:00 PM - 5:00 PM | 27.04 | 24.87 | 27.13 | -2.26 | 2.02 |
|  | (2.66) | (2.70) | (2.63) | (1.12) |  |
| 5:00 PM - 6:00 PM | 24.42 | 21.34 | 24.54 | -3.20 | 8.67 |
|  | (2.62) | (0.062) | (2.60) | (0.37) |  |

Note: The critical value for two-tail t test at 5% significance is 1.97.

In summary, workers paid by piece rate arrangements have lower hourly heat exposure than workers paid by hourly wages. Workers paid by piece rate arrangements have shorter exposure in the afternoon hours, when the heat is high. On average, workers paid by piece rate workers experience a cooler working environment during the same time of the day. Note that all the workers were observed in the summers of 2014 and 2015. The distribution of workers paid by different pay arrangements are not significantly different across different months or different crops.

# Methods

In many different fields including political science, economics, and statistics, studies have documented the reliability and usefulness of propensity score (Ho et al., 2017; Imbens and Wooldridge, 2009; Stuart, 2010). There are four common ways of using propensity scores (PS): PS matching, PS weighting, PS subclassification, and regression on the PS. Austin (Austin, 2009) finds that propensity matching has better performance in terms of eliminating a greater degree of the systematic differences between treated and untreated groups than the other three methods using both empirical and simulation studies.

The basic regression more in the article (Equation (1)) may fail to identify the effects of piece-rate wage policy on the worker’s response to heat exposure if the model fails to control some variables that affect both the workers’ likelihood of receiving piece-rate wage and workers’ reaction to heat exposure. The selection bias comes mainly from two potential sources. First, due to lack of data, our model probably omits variables that correlate to the attainment of piece-rate wage and worker’s response to heat exposure in the workplace. For example, our dataset does not have a physical strength variable. Physically strong workers more likely prefer piece-rate wages to hourly wages in order to obtain more income. Also, compared to physically weak workers, physically strong workers may be more tolerant of heat exposure and hence work more intensively than physically weak workers. Second, the linear model in Equation (1) may fail to specify the true relationship between the dependent variable (i.e., worker’s effort in the workplace) and the covariates. However, the covariates probably associate with the dependent variable nonlinearly. Many papers have documented that propensity score matching is less dependent on model specification (Ho et al., 2007).

To control the selection bias, we apply propensity score matching. (We do not apply the well-known approach of Heckman (1979) because we lack a set of instruments to effectively apply the specification error method. Propensity score is widely used to control potential selection bias. The propensity score was introduced by Rosenbaum and Rubin (Rosenbaum and Rubin, 1983); if the selection bias disappears conditional on the observed worker and job characteristics, the selection bias also disappears conditional on the propensity scores constructed by the observed characteristics. In our study, the selection bias in the linear regression model will disappear if we match each piece-rate-waged worker with an hourly-waged worker so that all the observed and unobserved aspects of the workers are identical. However, we cannot perfectly match the workers due to unobserved characteristics. As a second-best approach, we use the observed characteristics to match workers as precisely as we can. Using a sample with precisely matched workers possibly solves the selection bias derived from the model misspecification. Moreover, if the selection bias mostly comes from the model misspecification, we possibly get close to causal inference with matched workers. To clarify, in the rest of this chapter, we use the term “causal impact” under the assumption that our matching strategy successfully reduces the selection bias enough to explore the causation effect of piece rate arrangements.

Our goal is to match the workers paid by piece rate arrangements with the workers paid by hourly wages, using observed worker and job characteristics. One-to-one matching in terms of all the potential linear and nonlinear combinations of observed characteristics would be ideal. However, we have several categorical variables, which sharply raise the number of the combinations. Furthermore, the number of the combinations rises as we consider higher order effects of the same variables. In the current dataset, we observe each worker for only one day, and there are 575 workers in the dataset. The dataset we have does not allow a match with interactions of all observed variables, i.e., matching a worker paid by piece rate arrangements with a worker paid by hourly wage with all available observables. Instead, we use propensity scores to match the workers paid by piece rate arrangement approximately to the workers paid by hourly wages. The propensity score is the probability of workers receiving piece rate wage, given the observed worker and job characteristics.

There are some trade-offs when using the matched sample instead of the full sample. The matching step keeps workers who are similar to workers who were already paid by piece rate arrangements, and filters out workers who were paid by hourly wages and were unlikely being paid by piece rate arrangements. Hence the results of regression using the matches samples are showing the effect of heat exposure on the selected workers, rather than the effect on all workers that were observed in the full sample. Furthermore, workers in the matched sample have similar ages and hire types, and hence we do not have enough variation to identify the impacts of age and hire type when using the matched sample. For example, workers paid by piece rate arrangements tend to be younger and hired by contractors than the average worker paid by hourly wages arrangements. In the full sample estimate, we find the gender, age and hire type of workers significantly influence workers’ effort. Female workers tend to have lower effort than male workers. Older workers tend to have lower effort than young workers. Workers hired by contractors tend to have higher physical effort. In the matching step, workers with similar age and hire types are matched across the two pay arrangements (See S1 Results for the propensity score matching) such that the variation in age and hire type becomes too small to identify the estimate.

There are other approaches to address selection bias, such as synthetic control or Heckman’s framework for regression specification errors that is common in labor economics Heckman (1979). Abadie, Diamond, and Hainmueller (2010) introduced synthetic control methods to estimate the average treatment effect of tobacco control programs in California. The approach uses a weighted average of control units to construct the counterfactual for the treated group, with explicit relative contribution of each control unit to the counterfactual of interest. However, the performance of synthetic control in terms of selection bias control highly depends on a good match between treatment and control groups in the pre-treatment period. In the dataset that we analyze, we do not observe any pre-treatment period for workers, and each worker was only observed for one day without repeated observation.

# The results for the first step of Propensity Score matching

Table S5 shows the coefficient estimates for the logistic selection equation, Equation (2), using both logistic and linear regression. The coefficient estimates of the logistic regression could be interpreted as log-odds ratio. The coefficient estimates of the linear regression could be interpreted as probability. The signs of the estimates from both methods are consistent. Factors that decrease the probability of being paid by piece rate includes being female, large BMI value (overweight or obese), older, harvesting low and interviewed in August, compared to the base case that is directly hired male, working on pruning in June with an age of 36.5, and BMI 28.3 (overweight).

# Table S5. The coefficients and standard error of estimated Equation 1A for workers who are operating tree pruning/thinning and harvesting (Values in parentheses are standard errors).

|  | Logistic regression coef. | Linear regression coef. |
| --- | --- | --- |
| Female | -0.32 | -0.067 |
|  | (0.36) | (0.058) |
| BMI deviation from mean | -0.045 | -0.0069 |
|  | (0.038) | (0.0061) |
| Age deviation from mean | -0.014 | -0.0024 |
|  | (0.016) | (0.0026) |
| Hired as contractor | 0.69 | 0.11 |
|  | (0.40) | (0.066) |
| Month: July | 0.52 | 0.14 |
|  | (0.92) | (0.16) |
| Month: Aug | -1.81 | -0.32 |
|  | (0.98) | (0.16) |
| Month: Sep | 1.85 | 0.37 |
|  | (0.93) | (0.16) |
| Month: Oct | 16.86 | 0.40 |
|  | (1086.71) | (0.19) |
| Task: Harvest-low | -0.88 | -0.22 |
|  | (0.53) | (0.091) |
| Task: Harvest-high | 1.14 | 0.17 |
|  | (0.44) | (0.069) |
| Base case^b^ | 0.80 | 0.63 |
|  | (1.57) | (0.26) |
| Pseudo R-square^c^ | 0.30 | 0.35 |
| Number of observations | 243 ^a^ | 243 |

Note: ^a^ One worker (out of 244 workers) was excluded in the logistic regression due to missing hiring type information. ^b^ The base case is directly hired male, working on pruning in June with an age of 36.5 (average age of 244 workers), and BMI 28.3 (average BMI of 244 workers). ^c^ The R-square for the logistic regression is computed as McFadden’s Pseudo R square.

The projected probability of piece rate ranges from 0.02 to 1. If we use 0.5 as a cutoff point to project that the worker with a probability of piece paid by piece rate greater than 0.5 “is paid by piece rate arrangements,” and otherwise the worker “is paid by hourly wages.” The accuracy rate, that is the percentage of workers that have correct projected pay schemes based on the probability projection from logistic regression, is 76%. The accuracy rate is computed as

$$accurate=\frac{1}{243}(sum\left( workers paid by piece rate have prob.>0.5 \right)$$

$+ sum\left( workers paid by hourly rate have prob.<0.5 \right))$

Figure S1 shows the comparison between the projected probability of being paid by piece rate and workers’ actual pay arrangements. The left panel of Figure S1 shows the probability of being paid by piece rate for workers who were actually paid by hourly rate. Most of the workers who were paid by hourly rate have a probability less than 0.25 of being paid by piece rate arrangements. The right panel of Figure S1 shows the probability of being paid by piece rate for workers who were actually paid by piece rate. Most of the workers have a probability higher than 0.5. About 13 workers have a probability greater than 0.975.

# Figure S1. Comparison between the projected probability of piece rate arrangements and workers’ actual pay arrangement.


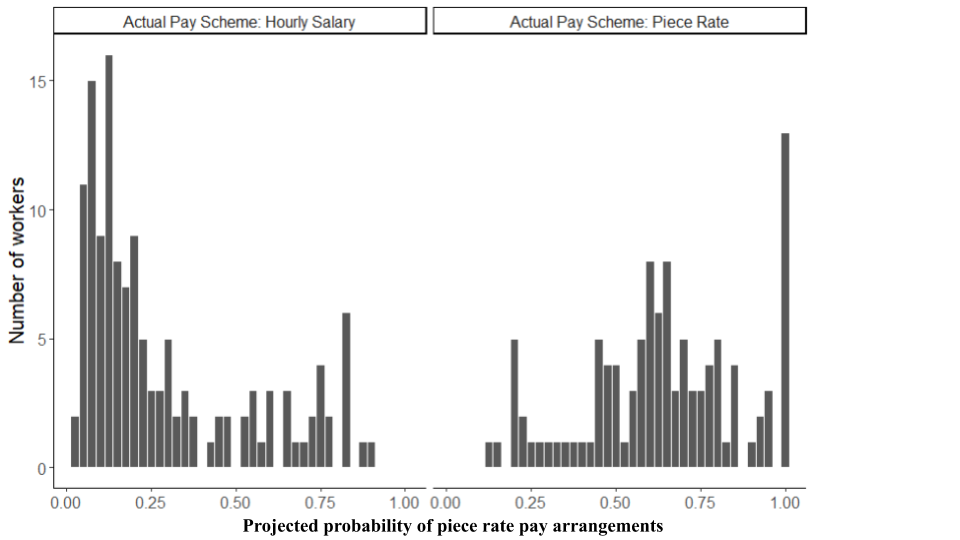


Note: There are a total of 243 workers with projected probability of being paid by piece rate. One worker with missing information on hiring type was excluded in both regression and projection.

Table S6 shows the summary statistics of 244 workers operating tree pruning/thinning and harvesting tasks before the propensity matching, and Table S7 shows the summary statistics of 216 workers after matching using propensity score. Before propensity matching, workers paid by piece rate and hourly wage rate are statistically different in terms of effort level, heat exposure, shift length, age, and BMI, and gender as shown in Table S6. After propensity matching, Table S7 shows that the only statistically significant differences between workers across different pay arrangements are the effort level, heat exposure, and shift length. The demographic differences are no longer statistically significant across workers paid by piece rate and hourly wage rate.

# Table S6: Summary statistics of worker effort, heat exposure and worker demographic characteristics for 244 workers operating tree pruning/thinning and harvesting.

|  |  | All workers | | Workers paid by piece rate | | Workers paid by hourly rate | | Test |
| --- | --- | --- | --- | --- | --- | --- | --- | --- |
| Numerical Variables | Unit | Number of obs. | mean  (s.d.) | Number of obs. | Mean  (s.d.) | Number of obs. | Mean  (s.d.) | t-score statistic ^e^ |
| hourly METs^a^ | kcal/h | 2653 | 1.90 | 1138 | 2.00 | 1515 | 1.82 | 7.10 |
|  |  |  | (0.64) |  | (0.65) |  | (0.62) |  |
| HourlyWBGT^b^ | ˚C | 2653 | 26.47 | 1138 | 22.63 | 1515 | 24.10 | 8.03 |
|  |  |  | (4.78) |  | (4.54) |  | (4.86) |  |
| Shift length | H:M | 244 | 8:03 | 109 | 7:16 | 135 | 8:40 | 9.93 |
|  |  |  | (1:17) |  | (1:01) |  | (1:09) |  |
| Age | Year | 244 | 36.50 | 109 | 35.12 | 135 | 38.50 | 2.33 |
|  |  |  | (11.41) |  | (10.99) |  | (11.57) |  |
| Body Mass Index^c^ | Index | 244 | 28.34 | 109 | 27.66 | 135 | 28.89 | 2.17 |
|  |  |  | (4.52) |  | (4.00) |  | (4.85) |  |
| Categorical Variables |  | All workers | | Workers paid by Piece Rate | | Workers paid by hourly rate | | Chi-squared statistic ^f^ |
|  |  | Number of obs.  Share % | | Number of obs.  Share % | | Number of obs.  Share % | |  |
| All |  |  | | 109 | | 135 | |  |
|  |  |  | | 44.7% | | 55.3% | |  |
| Gender |  |  |  |  |  |  |  | 5.94 |
| Male |  |  | 153 | 78 | | 75 | |  |
|  |  |  |  | 32.0% | | 30.7% | |  |
| Female |  |  | 91 | 31 | | 60 | |  |
|  |  |  |  | 12.7% | | 24.6% | |  |
| Hire Type^d^ |  |  |  |  |  |  |  | 0.34 |
| Direct Hire |  |  | 87 | 36 | | 51 | |  |
|  |  |  |  | 14.8% | | 20.9% | |  |
| Contractor |  |  | 156 | 72 | | 84 | |  |
|  |  |  |  | 29.5% | | 34.4% | |  |

Notes: ^a^ The range of the average metabolic equivalents (METs) from 1 to 2 is similar to the range of energy expenditure from sedentary activities such as sitting (Mitchell et al., 2018). ^b^ The calculation of hourly Wet Bulb Globe Temperature (WBGT) could be found in Section 3. ^c^ Based on World Health Organization BMI classification, an individual is considered 1) normal if 18.5≤BMI<25, 2) overweight if 25≤BMI<30, 3) obese if BMI>30. ^d^ Four workers have no hire type information, with two paid by piece rate and two workers paid by hourly wage rate. ^e^ T-score statistic greater than 1.96 implies statistical significance in different mean values. ^f^ Chi-square statistic greater than 3.84 implies correlation between pay type and gender (male or female) or hire type (contractor or direct hire).

# Table S7. Summary statistics of worker effort, heat exposure and worker demographic characteristics for 216 workers operating pruning/thinning and harvesting with propensity score matching.

|  |  | All workers | | Workers paid by piece rate | | Workers paid by hourly rate | | Test |
| --- | --- | --- | --- | --- | --- | --- | --- | --- |
| Numerical Variables | Unit | Number of obs. | mean  (s.d.) | Number of obs. | Mean  (s.d.) | Number of obs. | Mean  (s.d.) | t-score statistic ^e^ |
| hourly METs^a^ | kcal/h | 2343 | 1.91 | 1123 | 2.01 | 1219 | 1.83 | 6.83 |
|  |  |  | (0.64) |  | (0.65) |  | (0.62) |  |
| Hourly WBGT^b^ | ˚C | 2343 | 23.32 | 1123 | 22.62 | 1219 | 23.97 | 6.96 |
|  |  |  | (4.75) |  | (4.54) |  | (4.84) |  |
| Shift length | H:M | 216 | 7:59 | 108 | 7:17 | 108 | 8:42 | 9.24 |
|  |  |  | (1:20) |  | (1:05) |  | (1:09) |  |
| Age | Year | 216 | 35.63 | 108 | 35.27 | 108 | 35.98 | 0.47 |
|  |  |  | (10.97) |  | (10.93) |  | (11.05) |  |
| Body Mass Index^c^ | Index | 216 | 27.91 | 108 | 27.71 | 108 | 28.12 | 0.71 |
|  |  |  | (4.26) |  | (3.98) |  | (4.53) |  |
| Categorical Variables |  | All workers | | Workers paid by Piece Rate | | Workers paid by hourly rate | | Chi-squared statistic ^f^ |
|  |  | Number of obs.  Share % | | Number of obs.  Share % | | Number of obs.  Share % | |  |
| All |  |  | | 108 | | 108 | |  |
|  |  |  | | 50% | | 50% | |  |
| Gender |  |  |  |  |  |  |  |  |
| Male |  |  | 146 | 78 | | 68 | | 1.71 |
|  |  |  |  | 36.1% | | 31.5% | |  |
| Female |  |  | 70 | 30 | | 40 | |  |
|  |  |  |  | 13.9% | | 18.5% | |  |
| Hire Type^d^ |  |  |  |  |  |  |  | 0.32 |
| Direct Hire |  |  | 77 | 36 | | 41 | |  |
|  |  |  |  | 16.7% | | 19.0% | |  |
| Contractor |  |  | 139 | 72 | | 67 | |  |
|  |  |  |  | 33.3% | | 31.0% | |  |

Notes: ^a^ The range of the average metabolic equivalents (METs) from 1 to 2 is similar to the range of energy expenditure from sedentary activities such as sitting (Mitchell et al., 2018). ^b^ The calculation of hourly Wet Bulb Globe Temperature (WBGT) could be found in Section 3. ^c^ Based on World Health Organization BMI classification, an individual is considered 1) normal if 18.5≤BMI<25, 2) overweight if 25≤BMI<30, 3) obese if BMI>30. ^d^ Four workers have no hire type information, with two paid by piece rate and two workers paid by hourly wage rate. ^e^ T-score statistic greater than 1.96 implies statistical significance in different mean values. ^f^ Chi-square statistic greater than 3.84 implies correlation between pay type and gender (male or female) or hire type (contractor or direct hire).

# Robustness checks

This section conducts two robustness checks of the estimates for the impact of piece rate arrangements on effort during periods of high heat exposure. First, we check the robustness of the estimates reported in the main text by using a subsample of workers who perform the harvesting task, and the pruning or thinning task. With the majority of the tasks that were paid by piece rate arrangements are harvesting, pruning and thinning, therefore, we select all workers who worked at these tasks and estimate the impact of piece rate arrangements and heat exposure for that subsample.

As a second robustness check, we use air temperature instead of the Wet Bulb Globe Temperature (WBGT) to approximate the heat exposure of the workers. We use the same econometric models discussed above to estimate the impact of pay arrangements interacted with air temperatures on effort. The WBGT is often considered a direct measure for heat exposure and is considered direct and precise because it mimics the heat buildup process with clothing and shade. However, air temperature data is more widely available than WBGT and is a useful check on our results.

## *2.1 Regression results using 244 workers on selected tasks in full sample*

We estimated the main Equation (1) using the 244 workers working on harvesting, pruning and thinning in the full sample without propensity score matching. Table S8 shows the regression estimates for the 244 workers (who were harvesting, pruning and thinning) without data matching. Based on Column (1) in Table S8, piece rate arrangements increase average worker effort by about 0.06 METs. When not controlling pay arrangement, in Column (2), workers respond to heat exposure nonlinearly with an inverse-U shaped response function. Based on Column (3), the hourly paid workers response to heat exposure is close to zero and the coefficients are not statistically significant. The piece rate workers respond to heat exposure non-linearly with an inverse U-shaped response function.

# Table S8. The impact of piece rate and heat exposure (measured in WBGT) on workers’ effort (units in 0.01 METs) based on regression results using all workers working on harvesting and thinning in the full sample (Values in parentheses are standard errors).

|  | Dependent variable: Metabolic rate | | |
| --- | --- | --- | --- |
|  | (1) | (2) | (3) |
| WBGT |  | 2.99 | -0.56 |
|  |  | (2.28) | (2.88) |
| WBGT^2 |  | -0.12 | -0.03 |
|  |  | (0.043) | (0.05) |
| Piece rate | 6.43 |  | -117.35 |
|  | (3.47) |  | (44.38) |
| Piece rate: WBGT |  |  | 13.63 |
|  |  |  | (3.92) |
| Piece rate: WBGT^2 |  |  | -0.34 |
|  |  |  | (0.086) |
| Time of day | 0.75 | 0.81 | 0.83 |
|  | (0.031) | (0.045) | (0.048) |
| Time of day ^2 | -0.00039 | -0.00040 | -0.00041 |
|  | (0.000017) | (0.000021) | (0.000022) |
| Female | -10.96 | -9.13 | -9.02 |
|  | (3.38) | (3.41) | (3.38) |
| BMI | -0.15 | -0.12 | -0.11 |
|  | (0.32) | (0.32) | (0.33) |
| age | -0.12 | -0.14 | -0.101 |
|  | (0.16) | (0.15) | (0.15) |
| Hired by contractors | 4.94 | 4.21 | 3.57 |
|  | (4.01) | (4.16) | (4.14) |
| Task and Month fixed effects | Yes | Yes | Yes |
| R-square (without fixed effects) (%) | 27.6 | 28.6 | 29.7 |
| R-square (with fixed effects) (%) | 30.4 | 31.3 | 32.4 |
| N. of workers | 243 | 243 | 243 |
| N. of worker hour | 2638 | 2638 | 2638 |

Notes: Other covariates include nonlinear hours of day, task types, age, BMI, hire type, gender and month of the year. The standard error is worker-ID cluster-robust standard error. Column (1) represents results from regression model without no heat exposure variables, while including the pay arrangement variable, and all fix effects. Column (2) represents results from regression model without the pay arrangement variable but including heat exposure variables and fixed effects. Column (3) includes both heat exposure variables, pay arrangement variables, their interactions, and all fixed effects.

In addition, we estimate Equation (1) with both heat exposure variables and pay arrangement variables for female and male workers separately using the 244 workers who were harvesting, pruning and thinning in the full sample. Table S9 shows the regression estimates separately for these 90 female workers and 153 male workers. Based on Column 1 and Column 4, the piece rate arrangements increase the metabolic rate by a similar amount, with an increase of about 0.05 METs for male and female workers who were harvesting, pruning and thinning. Neither the estimates for male or female workers are statistically significant. Based on Column 2 and Column 5, we find the male workers respond to heat exposure non-linearly with an inverse-U shaped response, similar to findings estimated using in Table S8 Column 3. The changes in effort for female workers are close to zero and are not statistically significant. Based on Column 3, the female workers paid by hourly wages have very small changes in effort when facing increasing heat exposure, while the female workers paid by piece rate arrangements have statistically significant nonlinear changes in their effort. For male workers, based on Column 6, both groups of workers paid by piece rate arrangements and hourly wages have nonlinear changes in their effort when facing increasing heat exposure.

# Table S9. The impact of piece rate pay arrangements and heat exposure (measured in WBGT) on female and male workers’ effort (units in 0.01 METs) based on regression results using workers working on harvesting and thinning in the full sample (Values in parentheses are standard errors).

|  | Dependent variable:  Female metabolic rate | | | Dependent variable:  Male metabolic rate | | |
| --- | --- | --- | --- | --- | --- | --- |
|  | (1) | (2) | (3) | (4) | (5) | (6) |
| WBGT |  | -0.52 | -0.10 |  | 8.92 | 2.97 |
|  |  | (3.10) | (3.85) |  | (3.29) | (5.10) |
| WBGT^2 |  | -0.041 | -0.035 |  | -0.26 | -0.12 |
|  |  | (0.053) | (0.068) |  | (0.069) | (0.11) |
| Piece rate | 4.51 |  | -71.73 | 4.80 |  | -97.49 |
|  | (5.69) |  | (56.97) | (4.08) |  | (65.52) |
| Piece rate: WBGT |  |  | 9.97 |  |  | 10.97 |
|  |  |  | (4.67) |  |  | (6.08) |
| Piece rate: WBGT^2 |  |  | -0.28 |  |  | -0.27 |
|  |  |  | (0.098) |  |  | (0.14) |
| Time of day | 0.82 | 0.89 | 0.91 | 0.71 | 0.76 | 0.78 |
|  | (0.044) | (0.065) | (0.065) | (0.043) | (0.055) | (0.059) |
| Time of day ^2 | -0.00042 | -0.00044 | -0.00045 | -0.00037 | -0.00038 | -0.00039 |
|  | (0.000022) | (0.000028) | (0.000029) | (0.000023) | (0.000025) | (0.000028) |
| BMI | 0.090 | 0.18 | 0.19 | -0.40 | -0.38 | -0.35 |
|  | (0.47) | (0.48) | (0.50) | (0.42) | (0.42) | (0.42) |
| age | 0.21 | 0.14 | 0.14 | -0.18 | -0.21 | -0.17 |
|  | (0.26) | (0.28) | (0.28) | (0.18) | (0.17) | (0.17) |
| Hired by contractors | 15.50 | 16.25 | 14.14 | 0.58 | -1.64 | -1.34 |
|  | (9.50) | (9.58) | (9.83) | (4.22) | (4.42) | (4.36) |
| Task and Month fixed effects | Yes | Yes | Yes | Yes | Yes | Yes |
| R-square (without fixed effects) (%) | 34.1 | 34.8 | 35.9 | 23.7 | 25.4 | 26.0 |
| R-square (with fixed effects) (%) | 38.6 | 39.3 | 40.3 | 26.9 | 28.6 | 29.1 |
| N. of workers | 90 | 90 | 90 | 153 | 153 | 153 |
| N. of worker hour | 1010 | 1010 | 1010 | 1628 | 1628 | 1628 |

Notes: Other covariates include nonlinear hours of day, task types, age, BMI, hire type, gender and month of the year. The standard error is worker-ID cluster-robust standard error. Column (1) and (4) represent results from regression model without no heat exposure variables, while including the pay arrangement variable, and all fix effects. Column (2) and (5) represent results from regression model without the pay arrangement variable but including heat exposure variables and fixed effects. Column (3) and (6) include both heat exposure variables, pay arrangement variables, their interactions, and all fixed effects.

In summary, the finding based on 244 workers who are harvesting, pruning and thinning are consistent with findings using a full sample of 571 workers, and findings using 216 workers based on propensity score matched data. It shows that piece rate arrangements increase workers' total efforts, but workers would decrease their effort before the heat exposure becomes too high. Comparing all the different alternative sample selections, the results from the propensity score matching are more preferred, which addresses selection bias, and helps support the interpretation of causal impact of pay arrangements on workers’ effort.

## *2.2 Alternative heat exposure: air temperature*

In this section, we use air temperature instead of WBGT to test the robustness of the impact estimate for heat exposure and piece rate arrangements on workers’ effort. Compared to air temperature, the WBGT takes into account temperature, humidity, wind speed, sun angle and cloud cover. The WBGT captures more closely workers’ body responses to heat exposure than air temperatures. By exploring the robustness of the results using air temperature instead of WBGT allows us to check similar patterns in workers’ effort when facing changes in heat exposure measured in air temperature. Similar results indicate robustness of the finding to different heat exposure metrics.

Table S10 shows the estimates of the impacts of heat exposure and piece rate arrangements on workers’ effort using a full sample without propensity score matching. In Table S10 Column 1, workers paid by piece rate arrangements have higher effort (4.60 METs/hour) than workers paid by hourly wages, after controlling task types, hiring type and workers characteristics. In Column 2, workers have nonlinear response to heat exposure, with an inverse U-shaped functional form. The marginal response of effort to heat exposure is

$\frac{\partial mets}{\partial AirT}= 4.31-0.208 AirT$.

In Column 3, workers paid by hourly wages have an inverse U-shaped response in effort when facing increasing air temperatures, with the marginal response of effort to heat exposure as

$\frac{\partial mets_{hour}}{\partial AirT}=4.70-0.22 AirT$.

Workers paid by piece rate arrangements have nonlinear response to increasing air temperatures with an inverse U-shaped functional form. The marginal response of effort level to heat exposure is

$\frac{\partial mets_{piece}}{\partial AirT}= \left( 4.70+4.98 \right)-2\times(0.11+0.15) AirT$.

# Table S10. The impact piece rate pay arrangements and heat exposure (measured in air temperature) on workers’ effort (units in 0.01 METs) based on regression results using a full sample (Values in parentheses are standard errors).

|  | Full sample | | |
| --- | --- | --- | --- |
|  | (1) | (2) | (3) |
| Air temperature |  | 4.31 | 4.70 |
|  |  | (0.94) | (1.08) |
| Air temperature^2 |  | -0.104 | -0.11 |
|  |  | (0.017) | (0.019) |
| Piece rate | 4.60 |  | -27.94 |
|  | (3.09) |  | (23.96) |
| Piece rate * Air temperature |  |  | 4.98 |
|  |  |  | (2.11) |
| Piece rate* Air temperature^2 |  |  | -0.15 |
|  |  |  | (0.047) |
| Time of day | 0.55 | 0.56 | 0.56 |
|  | (0.017) | (0.022) | (0.022) |
| Time of day ^2 | -0.00028 | -0.00027 | -0.00027 |
|  | (0.0000086) | (0.000010) | (0.0000099) |
| Female | -13.75 | -13.45 | -13.65 |
|  | (2.94) | (2.96) | (2.96) |
| BMI | -0.22 | -0.22 | -0.20 |
|  | (0.21) | (0.22) | (0.22) |
| age | -0.25 | -0.23 | -0.22 |
|  | (0.096) | (0.095) | (0.097) |
| Hired by contractors | 5.69 | 5.87 | 5.62 |
|  | (2.65) | (2.62) | (2.69) |
| Task and Month fixed effects | Yes | Yes | Yes |
| R-square (without fixed effects) (%) | 21.1 | 22.0 | 22.6 |
| R-square (with fixed effects) (%) | 29.1 | 29.9 | 30.4 |
| N. of workers | 571 | 571 | 571 |
| N. of worker hour | 6603 | 6603 | 6603 |

Notes: Other covariates include nonlinear hours of day, task types, age, BMI, hire type, gender and month of the year. The standard error is worker-ID cluster-robust standard error. Column (1) represents results from regression model without no heat exposure variables, air temperature, while including the pay arrangement variable, and all fix effects. Column (2) represents results from regression model without the pay arrangement variable, but including heat exposure variables and fixed effects. Column (3) includes both heat exposure variables, pay arrangement variables, their interactions, and all fixed effects.

We estimate the differences in workers’ effort between workers paid by different pay arrangements as $\beta_{1}+\beta_{4}AirT_{ih}+\beta_{5}AirT_{ih}^{2}$ . Using the coefficient estimate from Column 3, we have:

$\Delta_{full}=-27.94+4.98 AirT-0.15 AirT^{2}$.

Figure S2 shows the differences in effort between workers paid by different pay arrangements in response to air temperatures estimated as $\Delta_{full}$. Based on Figure S2, workers are exerting more effort when the air temperature ranges from 12.81˚C to 24.13˚C. When the air temperature exceeds 29.48˚C, workers paid by piece rate arrangements exerts less effort than workers paid by hourly wage rate.

# Figure S2. Differences in effort between workers paid by piece rate and hourly wage arrangements in response to air temperature based on coefficients in Table S10.


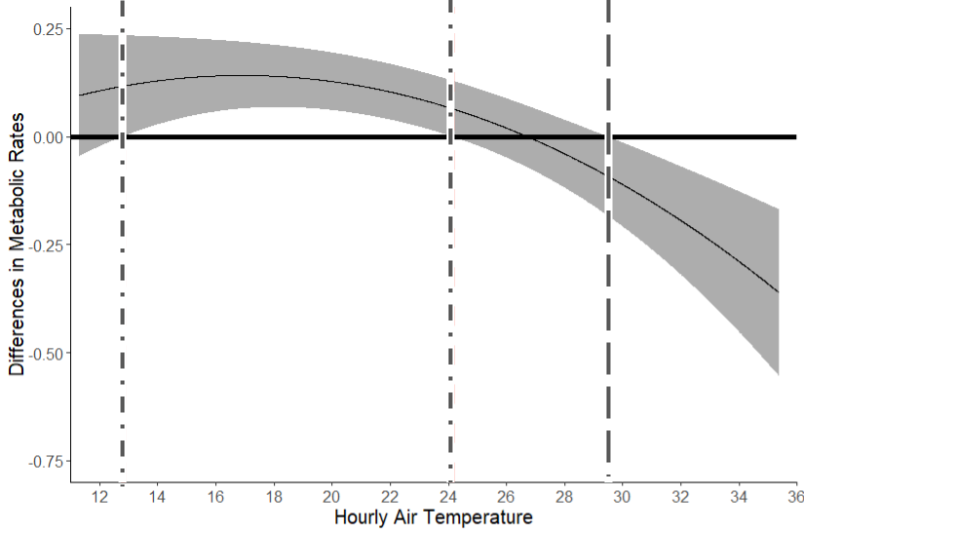


Note: The dot-dashed lines are the cut-off points of hourly WBGT where the estimated 95% confidence interval of the differences in metabolic rates between workers paid by piece rate and hourly rate is above zero. The dashed lines are the cut-off points of hourly WBGT where the estimated 95% confidence interval of the differences in metabolic rates between workers paid by piece rate and hourly rate is below zero.

Table S11 shows the regression results using propensity score matched samples. Notice that changing the heat exposure measure from WBGT to air temperature does not change the propensity matching steps because the heat exposure variable is not used in the matching process. In Table S11 Column 1, workers paid by piece rate arrangements have higher effort (6.98 METs/hour) than workers paid by hourly wages, after controlling tasks types, hiring type and workers characteristics. In Column 2, workers have a nonlinear response to heat exposure, with an inverse U-shaped functional form. The marginal response of effort to heat exposure is

$\frac{\partial mets}{\partial AirT}= 1.24-0.208 AirT$.

However, the coefficient estimates for air temperatures are statistically insignificant. In Column 3, workers paid by hourly wages shows a U-shaped effort response to increasing air temperatures, with the marginal response of effort level to heat exposure as

$\frac{\partial mets_{hour}}{\partial AirT}=-3.71+0.106 AirT$.

The coefficients estimated for workers paid by piece rate arrangements show nonlinear responses to increasing air temperature with an inverse U-shaped functional form. The marginal response of effort level to heat exposure is

$\frac{\partial mets_{piece}}{\partial AirT}= \left( 12.12-3.71 \right)-2\times(0.29-0.053) AirT$.

Using the coefficient estimate from Column 3, we estimate the differences in workers’ effort between workers paid by different pay arrangements as

$$\Delta_{matched}=-107.97+12.12 AirT-0.29 AirT^{2}$$

# Table S11. The impact of piece rate arrangements and heat exposure (measured in air temperature) on workers’ effort (units in 0.01 METs) based on regression results using a matched sample (Values in parentheses are standard errors).

|  | Full sample | | |
| --- | --- | --- | --- |
|  | (1) | (2) | (3) |
| Air temperature |  | 1.24 | -3.71 |
|  |  | (1.91) | (2.88) |
| Air temperature^2 |  | -0.059 | 0.053 |
|  |  | (0.040) | (0.058) |
| Piece rate | 6.98 |  | -107.97 |
|  | (3.52) |  | (36.75) |
| Piece rate: Air temperatue. |  |  | 12.12 |
|  |  |  | (3.27) |
| Piece rate: Air temperature^2 |  |  | -0.29 |
|  |  |  | (0.071) |
| Time of day | 0.73 | 0.75 | 0.80 |
|  | (0.033) | (0.042) | (0.049) |
| Time of day ^2 | -0.00038 | -0.00038 | -0.00040 |
|  | (0.000018) | (0.000021) | (0.000025) |
| Female | -13.97 | -12.90 | -12.84 |
|  | (3.50) | (3.56) | (3.54) |
| BMI | -0.38 | -0.36 | -0.26 |
|  | (0.38) | (0.37) | (0.40) |
| age | -0.21 | -0.22 | -0.18 |
|  | (0.17) | (0.16) | (0.17) |
| Hired by contractors | 0.95 | -0.95 | -1.06 |
|  | 4.08 | 4.47 | 4.50 |
| Task and Month fixed effects | Yes | Yes | Yes |
| R-square (without fixed effects) (%) | 27.2 | 27.5 | 29.1 |
| R-square (with fixed effects) (%) | 30.2 | 30.5 | 32.0 |
| N. of workers | 216 | 216 | 216 |
| N. of worker hour | 2341 | 2341 | 2341 |

Notes: Other covariates include non-linear hour of day effects, task types, age, BMI, hire type, gender and month of the year. The standard error is worker-ID cluster-robust standard error. Column (1) represents results from regression model without no heat exposure variables, while including the pay arrangement variable, and all fix effects. Column (2) represents results from regression model without the pay arrangement variable, but including heat exposure variables and fixed effects. Column (3) includes both heat exposure variables, pay arrangement variables, their interactions, and all fixed effects.

Figure S3 shows the differences in workers’ effort in response to air temperature between workers paid by different pay arrangements, estimated as $\Delta_{matched}$. Based on Figure S3, workers are exerting more effort when the air temperature ranges from 15.48˚C to 26.72˚C. When the air temperature exceeds 31.19˚C, workers paid by piece rate arrangements exert less effort than workers paid by hourly wages.

# Figure S3. Differences in effort between workers paid by piece rate and hourly wage arrangements in response to air temperature based on coefficients in Table S11.


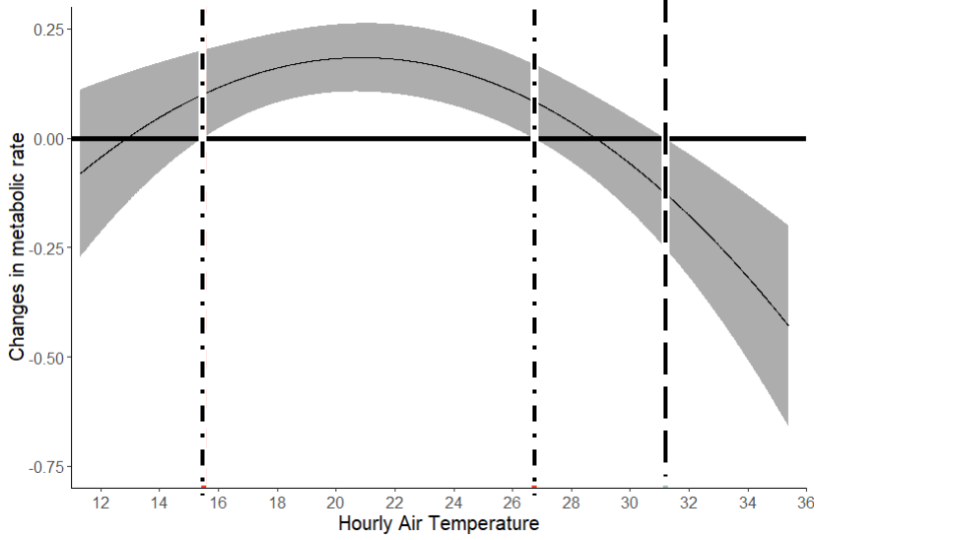


Differences in metabolic rates

Note: The dot-dashed lines are the cut-off points of hourly air temperature where the estimated 95% confidence interval of the differences in metabolic rates between workers paid by piece rate and hourly rate is above zero. The dashed lines are the cut-off points of hourly air temperature where the estimated 95% confidence interval of the differences in metabolic rates between workers paid by piece rate and hourly rate is below zero.

In summary, the main message when estimating heat exposure using air temperatures is similar to results using WBGT to measure heat exposure. Workers paid by piece rate pay arrangement decrease their effort before heat exposure becomes too high. The estimated impacts of piece rate arrangements on workers’ effort are larger when using the propensity score matched sample.

# References to S1 Appendix

1. Abadie A, Diamond A, Hainmueller J. Synthetic control methods for comparative case studies: Estimating the effect of California’s tobacco control program. Journal of the American Statistical Association. 2010 Jun 1;105(490):493-505.
2. Austin PC. The relative ability of different propensity score methods to balance measured covariates between treated and untreated subjects in observational studies. Medical Decision Making. 2009 Nov;29(6):661-77.
3. Bernard TE. Prediction of workplace wet bulb global temperature. Applied Occupational and Environmental Hygiene. 1999 Jan 1;14(2):126-34.
4. Crouter SE, Clowers KG, Bassett Jr DR. A novel method for using accelerometer data to predict energy expenditure. Journal of Applied Physiology. 2006 Apr;100(4):1324-31.
5. Gaspar AR, Quintela DA. Physical modelling of globe and natural wet bulb temperatures to predict WBGT heat stress index in outdoor environments. International Journal of Biometeorology. 2009 May;53(3):221-30.
6. Heckman JJ. Sample selection bias as a specification error. Econometrica: Journal of the Econometric Society. 1979 Jan 1:153-61.
7. Ho DE, Imai K, King G, Stuart EA. Matching as nonparametric preprocessing for reducing model dependence in parametric causal inference. Political Analysis. 2007;15(3):199-236.
8. Imbens GW, Wooldridge JM. Recent developments in the econometrics of program evaluation. Journal of Economic Literature. 2009 Mar;47(1):5-86.
9. Lemke B, Kjellstrom T. Calculating workplace WBGT from meteorological data: a tool for climate change assessment. Industrial Health. 2012;50(4):267-78.
10. Mitchell DC, Castro J, Armitage TL, Vega-Arroyo AJ, Moyce SC, Tancredi DJ, Bennett DH, Jones JH, Kjellstrom T, Schenker MB. Recruitment, methods and descriptive results of a physiologic assessment of latino farmworkers: the California heat illness prevention study (CHIPS). Journal of Occupational and Environmental Medicine. 2017 Jul;59(7):649.
11. Mitchell DC, Castro J, Armitage TL, Tancredi DJ, Bennett DH, Schenker MB. Physical activity and common tasks of California farm workers: California Heat Illness Prevention Study (CHIPS). Journal of Occupational and Environmental Hygiene. 2018 Dec 2;15(12):857-69.
12. Occupational Safety and Health Administration. Heat Illness Prevention Training Guide, A Lesson Plan for Employers. OSHA, 2011: 3437 - 2011.
13. Rosenbaum PR, Rubin DB. The central role of the propensity score in observational studies for causal effects. Biometrika. 1983 Apr 1;70(1):41-55.
14. Stuart EA. Matching methods for causal inference: A review and a look forward. Statistical Science: A Review Journal of the Institute of Mathematical Statistics. 2010 Feb 1;25(1):1.
15. Tonouchi M, Murayama K, Ono M. WBGT forecast for preventing heat strokes in Japan. In Sixth symposium on the urban environment. American Meteorological Society, Section PJ1 2006 Jan 30; (Vol. 1).

# Additional Tables

Table S1. Metabolic rate across tasks

| Task | Average mets |
| --- | --- |
| Multi-task | 1.9 |
| Irrigation | 1.8 |
| Ground pruner | 1.4 |
| Tree pruner | 1.9 |
| Harvest low | 1.7 |
| Harvest high | 2 |
| Hoeing and raking | 1.9 |
| Shoveling | 1.8 |
| Sorting | 1.4 |
| Carrying | 2 |
| Packing | 1.6 |
| Supervisor or driver | 1.7 |
| Nursery | 1.8 |

Table S2. Metabolic rate across crops

| Crops | Average Hourly Metabolic Rates |
| --- | --- |
| None | 1.8 |
| Melon | 1.7 |
| Peach | 2.0 |
| Grape | 1.9 |
| Pepper (Chilies and Jalapenos) | 1.4 |
| Cherry tomato | 1.9 |
| Tomato | 1.8 |
| Pistachio | 1.9 |
| Almond | 2.0 |
| Olive | 2.1 |
| Raspberry | 1.7 |
| Sweet Potato | 1.4 |
| Plum | 1.8 |
| Apricot | 1.8 |
| Nursery | 1.8 |
| Basil | 1.5 |
| Cotton | 1.3 |
| Squash | 1.7 |
| Flower | 1.6 |
| Garlic | 1.6 |
| Green Bean | 1.3 |
| Cucumber | 1.4 |
| Carrot | 2.0 |
| Corn | 1.8 |
| Walnut | 1.4 |
| Variety | 2.4 |
| Strawberry | 1.4 |
